# Race/ethnicity, disability, and antenatal depression in the United States: population-level insights from machine learning

**DOI:** 10.1016/j.pmedr.2026.103437

**Published:** 2026-03-07

**Authors:** Sangmi Kim, Moriah Chariz D. Cabadin

**Affiliations:** a Nell Hodgson Woodruff School of Nursing, Emory University, Atlanta, GA, USA; b Northside Hospital Forsyth, Cumming, GA, USA

**Keywords:** Antenatal depression, Race/ethnicity, Disability, Machine learning, PRAMS

## Abstract

**Objective:**

Women with intersecting identities, such as being both Black and disabled, face heightened risk of antenatal depression, yet few studies examine its nuanced mechanisms. To capture complex, interactive associations among risk factors, we applied explainable machine learning to predict antenatal depression and identify key predictors among non-Hispanic Black (NHB) and non-Hispanic White (NHW) women with and without disabilities in the U.S.

**Methods:**

Using 2019 Pregnancy Risk Assessment Monitoring System data merged with its disability supplement (*n* = 23,104), we developed random forest models for four subgroups defined by race/ethnicity and disability status. Model performance was evaluated using repeated 10-fold cross-validation with AUC. Variable importance and its stability were assessed through 50 refits of the final models with optimal hyperparameters.

**Results:**

NHW women and women with disabilities had higher rates of antenatal depression. Model performance was strong across subgroups (AUC: 0.79–0.89). Depression before pregnancy was the strongest predictor, followed by hypertension during pregnancy or smoking across subgroups. Having at least one disability contributed more strongly to prediction among NHB women, whereas depression screening was uniquely predictive among NHW women.

**Conclusions:**

Antenatal depression risk is shaped by women's intersecting identities. Nuanced subgroup differences should inform more targeted and equitable prevention strategies.

## Highlights

• Women with intersecting marginalized identities face higher antenatal depression risk.
• Machine learning reveals nuanced mechanisms of depression in intersecting identities.
• Black women reported lower antenatal depression than White women overall.
• Pre-pregnancy depression was the top predictor of antenatal depression.
• Predictors for antenatal depression varied by intersecting identities.

## 1 Introduction

Mental health is the leading cause of maternal mortality in the U.S., accounting for 22.7% of maternal deaths (Trost et al., 2022). Antenatal depression affects 10–30% among pregnant women in the U.S. (The American College of Obstetricians and Gynecologists, 2025; Mukherjee et al., 2016) Nevertheless, over 50% of pregnant women with depression were not treated (National Center for Chronic Disease Prevention and Health Promotion, 2022). Untreated antenatal depression increases the risk of maternal and infant morbidity and mortality (Dagher et al., 2021).

The risk of antenatal depression is disproportionately concentrated among women with marginalized identities, including racial/ethnic minorities and those with disabilities. The existing literature indicates an overall higher prevalence of antenatal depression among non-Hispanic Black (NHB) women than among non-Hispanic White (NHW) women (Mukherjee et al., 2016). However, there is conflicting evidence, with higher prevalence among NHW women (Sulley et al., 2023; Horner-Johnson et al., 2021; Bentley et al., 2025), depending on study design, setting, and sample characteristics.

Although historically underrepresented in research, the size of women with disabilities is not ignorable. In the U.S., approximately 12–18% of women of reproductive age have a type of disability that affects hearing, vision, cognition, mobility, self-care, and/or independent living (Horner-Johnson et al., 2021). Women with disabilities were up to three times more likely to experience depressive symptoms than those without during and after pregnancy (Alhusen et al., 2023a).

High rates of antenatal depression among NHB women and women with disabilities suggest that intersecting identities compound this risk. Intersectionality theory (Crenshaw, 2013) emphasizes how overlapping social identities interact to produce unique experiences of oppression, discrimination, and privilege. Black women with disabilities may face the combined effects of gendered racism and ableism, increasing their risk of discrimination, reduced access to care, and ultimately, antenatal depression. The U.S. has a history of stratified reproduction, wherein the fertility of White women is valued over that of women of color (Horner-Johnson et al., 2021). Longstanding narratives portray women with disabilities as asexual or unfit for motherhood, limiting access to high-quality preconception and prenatal care (Bentley et al., 2025).

Intersectionality highlights the importance of capturing the interactive and cumulative effects of multiple systems of oppression. To better account for the complex relationships between the diverse risk factors and antenatal depression, we found a need to employ a more flexible and robust analytical approach: explainable machine learning (ML). In contrast to linear regression models that rely on strict assumptions, such as linearity and independence and normality of errors, many ML algorithms can effectively model intricate interactions among variables without requiring them to be explicitly defined (Shah et al., 2019). Furthermore, explainable ML offers the advantage of quantifying variable importance (VI) to identify key predictors of antenatal depression as intervention targets.

While prior studies have examined antenatal depression among women of different racial and ethnic backgrounds or among women with disabilities, few have explored this risk at the intersection of race and disability status. To our knowledge, this is the first study to apply ML to investigate antenatal depression within this intersectional framework. This study aims to predict the risk of antenatal depression and identify key predictors among NHB and NHW women, stratified by disability status.

## 2 Methods

### 2.1 Data source

This secondary data analysis utilized data from the CDC's Pregnancy Risk Assessment Monitoring System (PRAMS) Phase 8 (2016–2022), linked with birth certificate data and the PRAMS disability supplement (Shulman et al., 2018). In 2019, PRAMS incorporated a disability supplement assessing impairments in vision, hearing, mobility, memory and concentration, activities of daily living, and communication. Because the disability supplement was administered only in 2019, our analysis was limited to that year's data.

Inclusion Criteria.

The study population consisted of those who 1) had information on their disability status and 2) identified themselves as NHB or NHW. Originally, the dataset included 67,710 individuals. We excluded those who had missing data on any of the six types of disability (*n* = 32,071) and those who were not NHB or NHW (*n* = 12,261). This sequential elimination process reduced the initial sample size to 23,378. After removing missing observations, the final sample size was 23,104.

Measures.

Of the original 491 variables available, 107 were initially selected based on prior literature identifying their associations with antenatal depression. Variables for survey year and U.S. state were also included in the analysis to consider potential cohort differences by birth year and geography. Only variables significantly associated with antenatal depression in bivariate analyses were retained in the final models: 47 and 40 for NHB women with and without disabilities, respectively; 58 and 51 for NHW women with and without disabilities, respectively.

### 2.2 Antenatal depression

The outcome variable was self-reported depression during pregnancy (yes/no).

### 2.3 Intersecting identities

We defined four subgroups based on maternal race and ethnicity and disability status. Race and ethnicity were categorized as NHB or NHW. Disability status was operationalized using a binary indicator reflecting the presence of at least one disability across six types—difficulty seeing, hearing, walking, remembering, with self-care, and communicating—regardless of severity. Each type of disability was originally measured on a four-point scale (“no difficulty” to “cannot do at all”). To capture disability more comprehensively, we created two additional variables: (1) the presence of each disability type (yes/no) and (2) a cumulative count of disabilities (range: 0–6). These variables were included as predictors in disability models.

### 2.4 Demographic and socioeconomic factors

These included maternal age, marital status, U.S. state, rurality, maternal education, health insurance coverage before and during pregnancy, annual household income, number of dependents, and receipt of benefits from the Special Supplemental Nutrition Program for Women, Infants, and Children (WIC) as a proxy for low income.

### 2.5 Psychosocial factors

These included acknowledgement of paternity, physical abuse by a husband/partner before and during pregnancy, physical abuse by an ex-husband/partner before and during pregnancy, and self-reported depression before pregnancy.

### 2.6 Medical factors

These factors included the number of pregnancy terminations in the past, infertility treatment, diabetes before and during pregnancy, hypertension before and during pregnancy, pre-pregnancy body mass index (BMI), gestational weight gain, mode of delivery (c-section, forceps, vacuum, and vaginal), and whether the mother or the infant was transferred for medical reasons.

### 2.7 Behavioral factors

These factors included pregnancy intention, the number of cigarettes smoked (before pregnancy, during first, second, and third trimester), the frequency of e-cigarettes use before and during pregnancy, and drinking in the last two years. Preconception care was assessed through access to pre-pregnancy healthcare, types of visits (e.g., visit for depression or anxiety), and specific topics discussed with providers (e.g., providers asked me if someone was hurting me emotionally or physically). Prenatal care (PNC) was evaluated by whether PNC was initiated during the first trimester, the total number of PNC visits, and adequacy of PNC based on Kessner and Kotelchuck indices. Similar to preconception care, specific topics discussed with providers during PNC visits were included.

### 2.8 Data analysis

**Variable Selection and Handling of Missing Data.** We removed three variables—NICU admission, type of healthcare coverage before pregnancy (“Other”), and alcohol use before pregnancy—whose degree of missing was 10% or greater (Appendix A) (Bennett, 2001). For the remaining variables, the proportion of missing observations ranged from 0% to 8.3%. We removed missing observations for the outcome variable, which is required to use ML for prediction. For the predictor variables, we conducted median imputation for numeric variables and assigned a code (“NA”) to missing observations for categorical variables with the expectation that our model would automatically learn what the missing patterns implied (Chollet et al., 2022).

**Descriptive Statistics.** We examined women's characteristics and their associations with antenatal depression, using Pearson's Chi-squared and Fisher's exact tests (for categorical variables) and Wilcoxon rank sum tests (for numeric variables).

**Classification Algorithms.** We built a random forest model (Wong et al., 2022) specific to four subgroups (two racial and ethnic groups [NHB and NHW] x two disability statuses [at least one disability and no disability]) to predict antenatal depression risk.

**Model Development.** The data was split 80/20 into training and testing sets, preserving the depression-to-no-depression ratio to address the data imbalance. To enhance model parsimony, we performed bivariate analyses of all 110 candidate variables and included only those significantly associated with antenatal depression among NHB and NHW women, respectively.

The predictors were pre-processed by normalizing the continuous variables and dummy-coding the categorical variables. Random forest has three hyperparameters (*mtry*, *trees*, and *min_n*), in which we set to generate 1000 different trees, leaving *mtry* (number of predictors to sample at each split) and *min_n* (minimum number of data points in a node) subject to tuning. We created a random grid whose size was as large as 300 (300 different combinations of *mtry* and *min_n*) using Latin hypercube sampling. Individual models with each possible combination of the hyperparameters were evaluated for their performance with repeated 10-fold cross-validation (CV). Final models were evaluated on the test set using the optimal hyperparameters. Discrimination (Van Calster et al., 2019) was assessed using the area under the receiver operating characteristic curve (AUC). Calibration (Van Calster et al., 2019) was examined with decile-based calibration plots, and probabilistic accuracy was quantified using the Brier score, with lower values indicating better agreement between predicted and observed outcomes.

**VI.** We estimated VI related to antenatal depression prediction from the training set via permutation testing (Altmann et al., 2010) and visualized the results for each subgroup. To assess VI stability, final models were refit using fixed optimal hyperparameters specific to each subgroup across 10-fold CV repeated five times. We summarized mean VI and predictor ranks, as well as the frequency of top 20 appearances across resamples. All data analyses were conducted using R version 4.4.1 (2024-06-14). This study was exempt from IRB review because it used publicly available, de-identified data.

## 3 Results

### 3.1 Subject characteristics and associations with antenatal depression

Overall, 22.5% of the study population reported antenatal depression. Relative to NHW women, NHB women exhibited more adverse socioeconomic, psychosocial, medical, and behavioral risk profiles. Despite these disparities, NHW women reported higher rates of antenatal depression than NHB women (23.5% vs. 20.8%). 45.5% of women across racial and ethnic groups had at least one disability (Table 1 & Appendix B).

**Table 1:** Characteristics of pregnant women by maternal race/ethnicity in 23 U.S. States and jurisdictions, 2019 pregnancy risk assessment monitoring system (short version).

	Overall N = 23,104^1^	Race and Ethnicity		*p*-value^2^
		Non-Hispanic Black *N* = 8,786^1^	Non-Hispanic White *N* = 14,318^1^	
Maternal Age	28.3 (5.7)	27.9 (5.9)	28.5 (5.5)	<0.001
At Least One Disability				<0.001
No	12,588 (54.5%)	5119 (58.3%)	7469 (52.2%)	
Yes	10,516 (45.5%)	3667 (41.7%)	6849 (47.8%)	
No. of Disabilities				<0.001
0	12,588.0 (54.5%)	5119.0 (58.3%)	7469.0 (52.2%)	
1	5964.0 (25.8%)	2135.0 (24.3%)	3829.0 (26.7%)	
2	2937.0 (12.7%)	988.0 (11.2%)	1949.0 (13.6%)	
3	1073.0 (4.6%)	355.0 (4.0%)	718.0 (5.0%)	
4	375.0 (1.6%)	113.0 (1.3%)	262.0 (1.8%)	
5	118.0 (0.5%)	47.0 (0.5%)	71.0 (0.5%)	
Difficulty Seeing				0.02
No	18,026 (78.0%)	6927 (78.8%)	11,099 (77.5%)	
Yes	5078 (22.0%)	1859 (21.2%)	3219 (22.5%)	
Difficulty Seeing				<0.001
1 = No difficulty	18,026 (78.0%)	6927 (78.8%)	11,099 (77.5%)	
2 = Some difficulty	4514 (19.5%)	1564 (17.8%)	2950 (20.6%)	
3 = Lot of difficulty	493 (2.1%)	246 (2.8%)	247 (1.7%)	
4 = Cannot do at all	71 (0.3%)	49 (0.6%)	22 (0.2%)	
Difficulty Hearing				<0.001
No	21,819 (94.4%)	8390 (95.5%)	13,429 (93.8%)	
Yes	1285 (5.6%)	396 (4.5%)	889 (6.2%)	
Difficulty Hearing				<0.001
1 = No difficulty	21,819 (94.4%)	8390 (95.5%)	13,429 (93.8%)	
2 = Some difficulty	1104 (4.8%)	316 (3.6%)	788 (5.5%)	
3 = Lot of difficulty	117 (0.5%)	35 (0.4%)	82 (0.6%)	
4 = Cannot do at all	64 (0.3%)	45 (0.5%)	19 (0.1%)	
Difficulty Walking				0.5
No	21,398 (92.6%)	8123 (92.5%)	13,275 (92.7%)	
Yes	1706 (7.4%)	663 (7.5%)	1043 (7.3%)	
Difficulty Walking				<0.001
1 = No difficulty	21,398 (92.6%)	8123 (92.5%)	13,275 (92.7%)	
2 = Some difficulty	1470 (6.4%)	542 (6.2%)	928 (6.5%)	
3 = Lot of difficulty	204 (0.9%)	95 (1.1%)	109 (0.8%)	
4 = Cannot do at all	32 (0.1%)	26 (0.3%)	6 (0.04%)	
Difficulty Remembering				<0.001
No	15,755 (68.2%)	6471 (73.7%)	9284 (64.8%)	
Yes	7349 (31.8%)	2315 (26.3%)	5034 (35.2%)	
Difficulty Remembering				<0.001
1 = No difficulty	15,755 (68.2%)	6471 (73.7%)	9284 (64.8%)	
2 = Some difficulty	6178 (26.7%)	1940 (22.1%)	4238 (29.6%)	
3 = Lot of difficulty	1129 (4.9%)	347 (3.9%)	782 (5.5%)	
4 = Cannot do at all	42 (0.2%)	28 (0.3%)	14 (0.1%)	
Difficulty with Self-care				0.01
No	22,377 (96.9%)	8544 (97.2%)	13,833 (96.6%)	
Yes	727 (3.1%)	242 (2.8%)	485 (3.4%)	
Difficulty with Self-care				<0.001
1 = No difficulty	22,377 (96.9%)	8544 (97.2%)	13,833 (96.6%)	
2 = Some difficulty	633 (2.7%)	198 (2.3%)	435 (3.0%)	
3 = Lot of difficulty	65 (0.3%)	20 (0.2%)	45 (0.3%)	
4 = Cannot do at all	29 (0.1%)	24 (0.3%)	5 (0.03%)	
Difficulty Communicating				<0.001
No	21,808 (94.4%)	8224 (93.6%)	13,584 (94.9%)	
Yes	1296 (5.6%)	562 (6.4%)	734 (5.1%)	
Difficulty Communicating				<0.001
1 = No difficulty	21,808 (94.4%)	8224 (93.6%)	13,584 (94.9%)	
2 = Some difficulty	1119 (4.8%)	468 (5.3%)	651 (4.6%)	
3 = Lot of difficulty	148 (0.6%)	70 (0.8%)	78 (0.5%)	
4 = Cannot do at all	29 (0.1%)	24 (0.3%)	5 (0.03%)	
Physical Abuse by Partner Before Pregnancy				<0.001
No	22,341 (96.7%)	8427 (95.9%)	13,914 (97.2%)	
Yes	763 (3.3%)	359 (4.1%)	404 (2.8%)	
Physical Abuse by Ex-Partner Before Pregnancy				0.01
No	22,211 (96.1%)	8410 (95.7%)	13,801 (96.4%)	
Yes	893 (3.9%)	376 (4.3%)	517 (3.6%)	
No. of Loss of Pregnancy	0.5 (1.0)	0.6 (1.0)	0.5 (0.9)	<0.001
Diabetes During Pregnancy				0.2
No	20,658 (89.4%)	7883 (89.7%)	12,775 (89.2%)	
Yes	2446 (10.6%)	903 (10.3%)	1543 (10.8%)	
Hypertension During Pregnancy				<0.001
No	18,357 (79.5%)	6766 (77.0%)	11,591 (81.0%)	
Yes	4747 (20.5%)	2020 (23.0%)	2727 (19.0%)	
Body Mass Index Before Pregnancy	28.0 (7.6)	29.1 (8.0)	27.4 (7.2)	<0.001
Maternal Weight Gain (lbs)	28.7 (16.3)	27.1 (16.9)	29.7 (15.8)	<0.001
Pregnancy Intention				<0.001
Later	4808 (21.1%)	2229 (25.7%)	2579 (18.3%)	
Not sure	4630 (20.3%)	2179 (25.1%)	2451 (17.4%)	
Not want	1902 (8.4%)	971 (11.2%)	931 (6.6%)	
Sooner	2736 (12.0%)	714 (8.23%)	2022 (14.3%)	
Then	8705 (38.2%)	2580 (29.7%)	6125 (43.4%)	
Unknown	323	113	210	
No. Cigarettes Before Pregnancy	1.7 (5.8)	0.9 (4.2)	2.3 (6.5)	<0.001
Ask me if I was feeling down or depressed				<0.001
No	5262 (22.8%)	1599 (18.2%)	3663 (25.6%)	
Yes	17,842 (77.2%)	7187 (81.8%)	10,655 (74.4%)	
No. of Prenatal Care Visits	11.1 (4.5)	10.6 (4.9)	11.3 (4.2)	<0.001
Ask if I was feeling down or depressed				<0.001
No	3474 (15.0%)	1071 (12.2%)	2403 (16.8%)	
Yes	19,630 (85.0%)	7715 (87.8%)	11,915 (83.2%)	
Depression Before Pregnancy				<0.001
No	17,904 (77.5%)	7197 (81.9%)	10,707 (74.8%)	
Yes	5200 (22.5%)	1589 (18.1%)	3611 (25.2%)	
Depression During Pregnancy				<0.001
No	17,910 (77.5%)	6961 (79.2%)	10,949 (76.5%)	
Yes	5194 (22.5%)	1825 (20.8%)	3369 (23.5%)	

1 n (%); Mean (SD).

2 Pearson's Chi-squared test; Wilcoxon rank sum test.

Women with at least one disability were more likely to report antenatal depression than those without (33.8% vs. 13%), with rates increasing alongside the severity and number of disabilities. Healthcare visits before pregnancy were associated with lower rates of antenatal depression, while being asked about violence experience and depression during these visits was associated with higher rates. 70.2% of women with depression before pregnancy likely experienced antenatal depression (Appendix C).

When considering both race and ethnicity and disability status, disparities between NHW and NHB women—higher rates of antenatal depression among the former—were further amplified among those with at least one disability across most maternal characteristics (Appendix D).

### 3.2 Model performance

Overall, the random forest models performed well, with AUC values ranging from 0.79 to 0.89. Models for NHW women or women with disabilities demonstrated stronger discrimination compared to their respective counterparts. However, models for women without disabilities had lower Brier scores, reflecting better calibration or closer alignment between predicted and observed outcomes (Table 2). Across four subgroups, calibration plots showed a tendency to slightly overpredict risk at the lower end of the predicted probability spectrum and underpredict risk at the higher end, although overall calibration was reasonable. Notably, among NHB women with disabilities, the model underestimated risk in a very high-risk subgroup, whereas among NHW women with disabilities, it overestimated risk in a similarly high-risk subgroup (Figs. S1–S4). Sensitivity analyses—combining individuals with and without disabilities and including disability status as a predictor—yielded performance comparable to the models stratified by disability status.

**Table 2:** Prediction accuracy of random forest models by race/ethnicity and disability status in 23 U.S. States and jurisdictions, 2019 pregnancy risk assessment monitoring system.

	Non-Hispanic Black		Non-Hispanic White	
	Area Under the Receiver Operating Characteristic Curve (95% CI)	Brier	Area Under the Receiver Operating Characteristic Curve (95% CI)	Brier
No Disability	0.79 (0.75, 0.84)^1^	0.08	0.86 (0.83, 0.89)^4^	0.08
Disability	0.85 (0.82, 0.88)^2^	0.14	0.89 (0.87, 0.91)^5^	0.13
Combined (sensitivity test)	0.85 (0.82, 0.87)^3^	0.12	0.89 (0.87, 0.90)^6^	0.10

CI = confidence interval.

1 Selected hyperparameters: *mtry* = 4, *min_n* = 39.

2 Selected hyperparameters: *mtry* = 10, *min_n* = 40.

3 Selected hyperparameters: *mtry* = 4, *min_n* = 39.

4 Selected hyperparameters: *mtry* = 11, *min_n* = 33.

5 Selected hyperparameters: *mtry* = 10, *min_n* = 10.

6 Selected hyperparameters: *mtry* = 9, *min_n* = 38.

### 3.3 VI

**Fig. 1:**
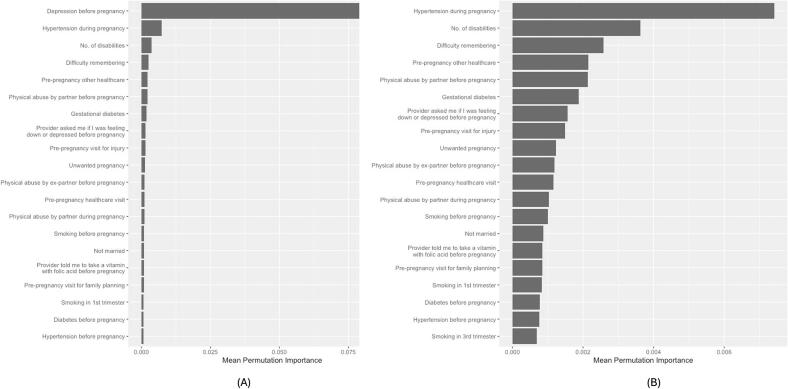
Top 20 predictors of antenatal depression among non-Hispanic Black women with at least one disability in 23 U.S. States and Jurisdictions, 2019 Pregnancy Risk Assessment Monitoring System. (A) Top 20 predictors including depression before pregnancy. Because depression before pregnancy had substantially higher importance, the scale is anchored to this variable, which compresses the relative magnitude of the remaining variables. (B) Top 19 predictors with depression before pregnancy removed to allow clearer visualization of differences in importance among the remaining variables. Variable importance values are unchanged across panels; only the scale differs.

Depression before pregnancy emerged as the strongest predictor of antenatal depression across four subgroups, consistently ranking among the top 20 predictors in all 50 model refits. Among NHB women *with* disabilities, hypertension during pregnancy ranked second (Fig. 1) and appeared in the top 20 in 100% of refits. Although the number of disabilities and difficulty remembering or concentrating ranked third and fourth, neither appeared in the top 20 in any refit.

**Fig. 2:**
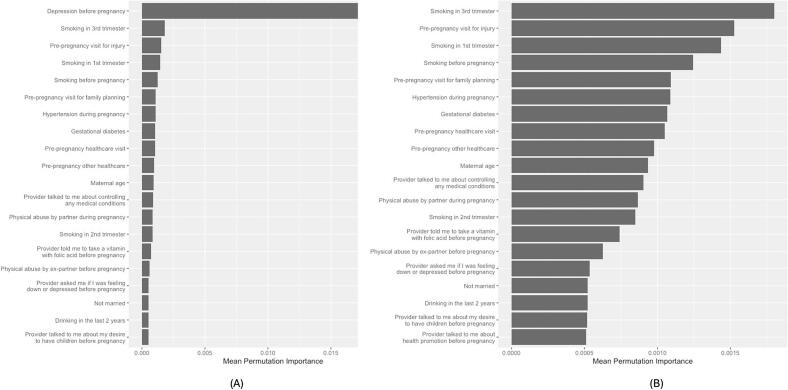
Top 20 predictors of antenatal depression among non-Hispanic Black women without disability in 23 U.S. States and Jurisdictions, 2019 Pregnancy Risk Assessment Monitoring System. (A) Top 20 predictors including depression before pregnancy. Because depression before pregnancy had substantially higher importance, the scale is anchored to this variable, which compresses the relative magnitude of the remaining variables. (B) Top 19 predictors with depression before pregnancy removed to allow clearer visualization of differences in importance among the remaining variables. Variable importance values are unchanged across panels; only the scale differs.

Among NHB women *without* disabilities, smoking in the third trimester ranked second (Fig. 2) and appeared in the top 20 in 74% of refits.

**Fig. 3:**
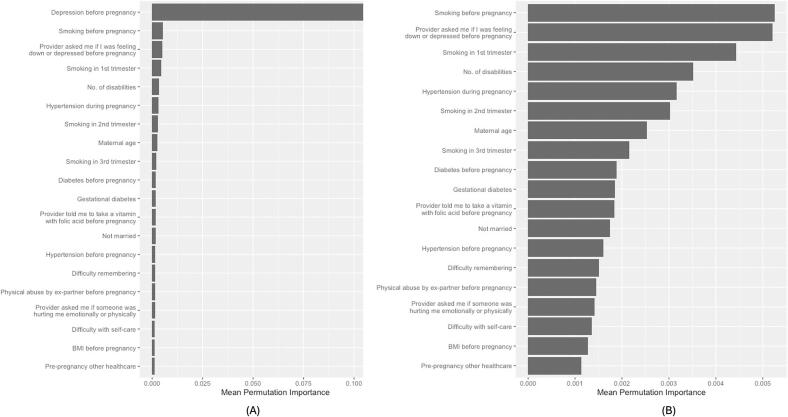
Top 20 predictors of antenatal depression among non-Hispanic White women with at least one disability in 23 U.S. States and Jurisdictions, 2019 Pregnancy Risk Assessment Monitoring System. (A) Top 20 predictors including depression before pregnancy. Because depression before pregnancy had substantially higher importance, the scale is anchored to this variable, which compresses the relative magnitude of the remaining variables. (B) Top 19 predictors with depression before pregnancy removed to allow clearer visualization of differences in importance among the remaining variables. Variable importance values are unchanged across panels; only the scale differs.

Among NHW women *with* disabilities, smoking before pregnancy and depression screening before pregnancy—being asked by a healthcare provider if they were feeling down or depressed during preconception care—followed depression before pregnancy in importance (Fig. 3). Smoking and depression screening before pregnancy appeared in the top 20 in 50% and 48% of refits, respectively.

**Fig. 4:**
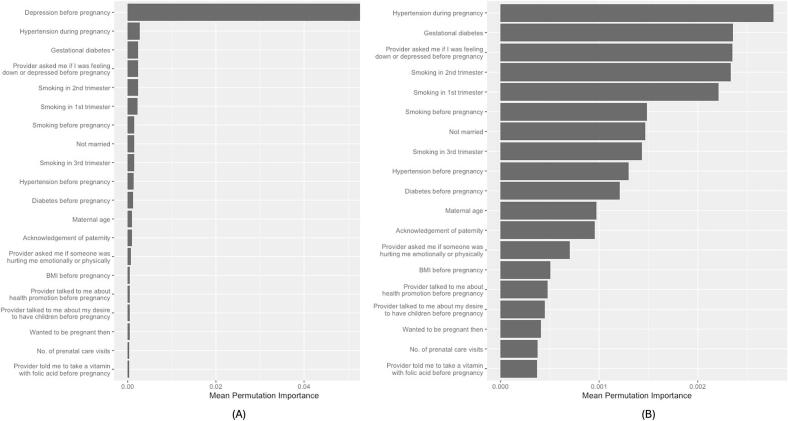
Top 20 predictors of antenatal depression among non-Hispanic White women without disability in 23 U.S. States and Jurisdictions, 2019 Pregnancy Risk Assessment Monitoring System. (A) Top 20 predictors including depression before pregnancy. Because depression before pregnancy had substantially higher importance, the scale is anchored to this variable, which compresses the relative magnitude of the remaining variables. (B) Top 19 predictors with depression before pregnancy removed to allow clearer visualization of differences in importance among the remaining variables. Variable importance values are unchanged across panels; only the scale differs.

Among NHW women *without* disabilities, hypertension during pregnancy ranked second (Fig. 4) and appeared in the top 20 in 64% of refits. Although smoking in the second trimester and depression screening before pregnancy ranked third and fourth, they appeared in the top 20 in only 14% and 10% of refits, respectively.

Although models combining individuals with and without disabilities showed considerable overlap with models stratified by disability status in VI (Figs. S5 and S6), they obscured nuanced differences in the type and magnitude of predictors across subgroups. Both combined models identified having at least one disability as a key predictor, ranking second in the NHB model and fourth in the NHW model.

## 4 Discussion

This study leveraged explainable ML and national surveillance data on pregnant women in the U.S. to predict the risk of antenatal depression and identify its key predictors across four subgroups: NHB and NHW women, with and without disabilities. Our findings address a critical gap in understanding Black-White disparities in antenatal depression by centering women with intersecting marginalized identities, particularly NHB women with disabilities. The analysis showed that the combined effect of having both marginalized identities on antenatal depression was greater than the individual effect of each identity. It also revealed both shared and subgroup-specific risk factors, offering insight into the nuanced mechanisms driving these disparities. The use of a random forest model enhanced this new evidence base by capturing complex, non-linear relationships and quantifying the extent to which each risk factor contributes to the prediction.

Consistent with prior research, our study found that women with disabilities reported higher rates of antenatal depression than those without (Alhusen et al., 2023). However, contrary to expectations and much of the existing literature (Gavin et al., 2011), NHW women in this study, regardless of disability status, were more likely to report antenatal depression than NHB women. While some studies have documented similar patterns (Cook et al., 2010, Loprinzi et al., 2012), the literature on racial and ethnic disparities in antenatal depression remains mixed (Mukherjee et al., 2016).

This finding is particularly striking given the structural disadvantages and psychosocial stressors disproportionately affecting Black women. We propose three interrelated explanations. First, underreporting of depressive symptoms may stem from cultural stigma and gendered racial ideologies, such as the Strong Black Woman (SBW) schema (Woods-Giscombe et al., 2016), which valorizes emotional restraint, self-sacrifice, and resilience. These expectations, coupled with the frequent use of disengagement or avoidance coping strategies (Lewis et al., 2017), may lead Black women to underrecognize or suppress depressive symptoms.

Second, our models did not account for experiences of racial discrimination, a known contributor to antenatal depression (Canady et al., 2008), potentially leading to underestimation of antenatal depression among NHB women.

Finally, the binary structure of the PRAMS antenatal depression measure may fail to capture the full spectrum of symptom severity. Fellenzer and Cibula (Fellenzer and Cibula, 2014) suggests that NHB women are more likely than their White counterparts to report severe depressive symptoms. Given that mild to moderate symptoms are more prevalent and more likely to go unrecognized, particularly when filtered through cultural scripts of strength and silence, our findings may reflect not a true absence of depressive symptoms, but rather their invisibility.

Despite overall strong discrimination, the divergence between discrimination and calibration and its differing patterns between NHB and NHW women with disabilities may reflect underlying heterogeneity or potential measurement error in antenatal depression assessment within disability subgroups (Van Calster et al., 2019), and suggests that further model refinement and validation are necessary before clinical application.

The key predictors of antenatal depression identified in this study align with the well-established risk factors. Depression before pregnancy was the strongest predictor (Yin et al., 2021), with hypertension during pregnancy (Mogos et al., 2019; Shay et al., 2020; Dachew et al., 2021) and smoking (Yin et al., 2021) also contributing substantially across four subgroups.

Depression screening before pregnancy was uniquely predictive among NHW women, particularly those with disabilities. In contrast, its lower predictive value among NHB women raises concerns about conventional screening tools, which may overlook culturally specific symptom profiles. Black women often experience and express depression through somatic symptoms and negative self-perceptions rather than depressed mood (Perez et al., 2023). Because PRAMS assesses whether a provider asked if the individual felt “down” or “depressed,” it may miss non-traditional or culturally nuanced expressions of depression, leading to underestimation among NHB women. While depression screening before pregnancy remains important for early intervention, this gap underscores the need for culturally responsive screening tools that better capture the symptomatology of NHB women (Alhusen et al., 2023, Perez et al., 2023).

In sensitivity analyses, having at least one disability ranked second most important predictor among NHB women and fourth among NHW women, suggesting a stronger relative contribution of disability to antenatal depression risk among NHB women. This pattern is notable given that both the prevalence and the number of disability were higher among NHW women, underscoring the amplified risk of antenatal depression among women with intersecting marginalized identities.

Several limitations warrant consideration. The PRAMS disability supplement was administered only in 23 states in 2019, limiting sample size and generalizability. To preserve statistical power and ensure comparability across states, our analysis was restricted to the PRAMS core questions administered uniformly across all participating states. Consequently, important variables known to influence mental health, like social support and perceived discrimination, were excluded, as they were only collected in a few states. The absence of these variables may have reduced the predictive accuracy among NHB and disabled subgroups. Finally, the self-reported nature of PRAMS data introduces limitations, including recall bias, social desirability bias, and response bias, which are particularly salient in the context of mental health and may contribute to underreporting of antenatal depression.

## 5 Conclusion

By applying explainable ML, this study uncovered nuanced mechanisms underlying antenatal depression at the intersection of race, ethnicity, and disability. As mental health conditions are the leading cause of maternal mortality in the U.S. (Trost et al., 2022) identifying intersectional drivers of antenatal depression is essential to inform more equitable preventive strategies. The lower rates of antenatal depression observed among NHB women in this study may not reflect a lower burden but rather entrenched structural barriers to recognizing, reporting, and treating antenatal depression, especially when disability is present. Addressing these barriers requires systemic efforts to confront gendered racism and ableism, along with universal, culturally responsive depression screening, expanding access to preventive care for chronic conditions, and smoking cessation support before and during pregnancy, especially for women who are NHB or disabled.

## Data statement

The data used in this study are unavailable to post, as access to the PRAMS dataset requires prior approval from the Centers for Disease Control and Prevention (CDC). At this time, the application portal for data access is temporarily closed. In accordance with the CDC's Data Sharing Agreement, only approved personnel are permitted to access and analyze the dataset.

## CRediT authorship contribution statement

**Sangmi Kim:** Writing – review & editing, Writing – original draft, Visualization, Validation, Supervision, Software, Resources, Project administration, Methodology, Investigation, Funding acquisition, Formal analysis, Data curation, Conceptualization. **Moriah Chariz D. Cabadin:** Writing – review & editing, Writing – original draft, Visualization, Investigation, Conceptualization.

## Funding

This work was supported by the National Institute of Nursing Research of the National Institutes of Health under Award Numbers K01NR019651 and R25NR021324. The authors have no competing financial interests or personal relationships that could have appeared to influence the work reported in this paper.

## Declaration of competing interest

The authors declare the following financial interests/personal relationships which may be considered as potential competing interests: Sangmi Kim reports financial support was provided by National Institute of Nursing Research. If there are other authors, they declare that they have no known competing financial interests or personal relationships that could have appeared to influence the work reported in this paper.

## Data Availability

The authors do not have permission to share data.

## Appendix A Supplementary data

Appendix A Image 1

Appendix B Image 2

Appendix C Image 3

Appendix D Image 4

Supplementary figures S1-S6 Image 5
