# Supplementary material for: Race/ethnicity, disability, and antenatal depression in the United States: population-level insights from machine learning
Source: Prev Med Rep. 2026 Mar 7;65:103437. doi: 10.1016/j.pmedr.2026.103437 (PMC12996995; doi:10.1016/j.pmedr.2026.103437)
Supplement: Supplementary file 1 — Appendix A [file mmc1.docx]

Appendix A. Frequency and Percentage of Missing Data for Selected Pregnancy Risk Assessment Monitoring System (PRAMS) Phase 8 Variables Among Pregnant Women, 23 U.S. States and Jurisdictions, 2019

|  | Variable | Label | Frequency | Percentage |
| --- | --- | --- | --- | --- |
| 1 | AB_NICU | Infant admitted to NICU | 23378 | 100 |
| 2 | TYP_WHAT | Health care type—other specified | 21218 | 90.8 |
| 3 | DRK8_3B | Drinking 3 months before pregnancy | 8035 | 34.4 |
| 4 | INCOME8 | Annual household income | 1935 | 8.28 |
| 6 | INC_NDEP | Number of income dependents | 706 | 3.02 |
| 7 | MOM_BMI | Pre-pregnancy body mass index (BMI) | 662 | 2.83 |
| 8 | PNC_1TRM | Prenatal care started in 1st trimester | 601 | 2.57 |
| 9 | PNC_VST | Number of prenatal care visits | 497 | 2.13 |
| 10 | KOTELCHUCK | Adequacy of prenatal care (Kotelchuck Index) | 484 | 2.07 |
| 11 | MOMLBS | Gestational weight gain (maternal pounds gained) | 467 | 2.00 |
| 13 | PGINTENT | Pregnancy intention | 337 | 1.44 |
| 14 | ECIG_3B_A | E-cigarette use before 3 months before pregnancy | 186 | 0.796 |
| 16 | ECIG_3L_A | E-cigarette use in the last 3 months | 162 | 0.693 |
| 18 | MAT_DEG | Maternal education level | 103 | 0.441 |
| 19 | CIG_1TRI | Cigarette use during 1st trimester | 102 | 0.436 |
| 20 | CIG_PRIOR | Cigarette use before pregnancy | 101 | 0.432 |
| 21 | CIG_3TRI | Cigarette use during 3rd trimester | 99 | 0.423 |
| 22 | CIG_2TRI | Cigarette use during 2nd trimester | 98 | 0.419 |
| 24 | OTH_TERM | Number of other pregnancy terminations | 40 | 0.171 |
| 25 | NCHS_URB_RUR2 | Urban vs. rural residence (NCHS classification) | 22 | 0.094 |
| 26 | INSNONE | I did not have any health insurance during the month before I got pregnant. | 18 | 0.077 |
| 27 | INFER_TR | Infertility treatment | 18 | 0.077 |
| 37 | MAT_TRAN | Mother transferred | 5 | 0.021 |
| 38 | YY4_DOB | Year of birth | 0 | 0 |
| 39 | STATE | U.S. state | 0 | 0 |
| 40 | MAT_AGE_PU | Maternal age | 0 | 0 |
| 41 | MARRIED | Marital status | 0 | 0 |
| 42 | PAT_ACK | Paternity acknowledgment | 0 | 0 |
| 43 | HISPANIC | Hispanic ethnicity | 0 | 0 |
| 44 | MRACE_BLK | Maternal race – Black | 0 | 0 |
| 45 | MRACE_WHT | Maternal race – White | 0 | 0 |
| 46 | DIFF_SEE | Have difficulty seeing | 0 | 0 |
| 47 | DIFF_HEAR | Have difficulty hearing | 0 | 0 |
| 48 | DIFF_WALK | Have difficulty walking | 0 | 0 |
| 49 | DIFF_REM | Have difficulty remembering | 0 | 0 |
| 50 | DIFF_CARE | Have difficulty with self-care | 0 | 0 |
| 51 | DIFF_COMM | Have difficulty communicating | 0 | 0 |
| 52 | PP_NONE | I did not have any health insurance to pay for my prenatal care. | 0 | 0 |
| 53 | MAT_WIC | Mother get WIC food during pregnancy? | 0 | 0 |
| 54 | PAB6HUS | Physical abuse by husband/partner before pregnancy | 0 | 0 |
| 55 | PAD6HUS | Physical abuse by husband/partner during pregnancy | 0 | 0 |
| 56 | PAB_XHUS | Physical abuse by ex-partner before pregnancy | 0 | 0 |
| 57 | PAD_XHUS | Physical abuse by ex-partner during pregnancy | 0 | 0 |
| 58 | BPG_DIAB8 | Diabetes before pregnancy | 0 | 0 |
| 59 | BPG_HBP8 | Hypertension before pregnancy | 0 | 0 |
| 60 | PG_GDB8 | Gestational diabetes | 0 | 0 |
| 61 | MORB_BP8 | Hypertension during pregnancy | 0 | 0 |
| 62 | DRK_2YRS | Drinking in the last 2 years | 0 | 0 |
| 63 | PRE_VIST | Pre-pregnancy healthcare visit | 0 | 0 |
| 64 | TYP_DOCT | Pre-pregnancy healthcare type – checkup with doctor | 0 | 0 |
| 65 | TYP_OBGN | Pre-pregnancy healthcare type – checkup with my OB/GYN | 0 | 0 |
| 66 | TYP_ILLN | Pre-pregnancy healthcare type – visit for illness | 0 | 0 |
| 67 | TYP_INJR | Pre-pregnancy healthcare type – visit for injury | 0 | 0 |
| 68 | TYP_BC | Pre-pregnancy healthcare type – visit for family planning/birth control | 0 | 0 |
| 69 | TYP_MH | Pre-pregnancy healthcare type – visit for depression or anxiety | 0 | 0 |
| 70 | TYP_DDS | Pre-pregnancy healthcare type – visit with dentist | 0 | 0 |
| 71 | TYP_OTHR | Pre-pregnancy healthcare type – other | 0 | 0 |
|  | During pre-pregnancy healthcare visits, a healthcare worker did: | | |  |
| 72 | PRE_VIT | Tell me to take vitamin with folic acid | 0 | 0 |
| 73 | PRE_WT | Talk to me about maintaining a healthy weight | 0 | 0 |
| 74 | PRE_MORB | Talk to me about controlling any medications such as diabetes or high blood pressure | 0 | 0 |
| 75 | PRE_KIDS | Talk to me about my desire to have or not have children | 0 | 0 |
| 76 | PRE_PRBC | Talk to me about using birth control to prevent pregnancy | 0 | 0 |
| 77 | PRE_HLTH | Talk to me about how I could improve my health before a pregnancy | 0 | 0 |
| 78 | PRE_STI | Talk to me about sexually transmitted infections | 0 | 0 |
| 79 | PRE_SMK | Ask me if I was smoking cigarettes | 0 | 0 |
| 80 | PRE_ABUS | Ask me if someone was hurting me emotionally or physically | 0 | 0 |
| 81 | PRE_MHDP | Ask me if I was feeling down or depressed | 0 | 0 |
| 82 | PRE_WORK | Ask me about the kind of work I do | 0 | 0 |
| 83 | PRE_HIVT | Test me for HIV | 0 | 0 |
| 84 | KESSNER | Adequacy of prenatal care (Kessner Index) | 0 | 0 |
|  | During prenatal care visits, a healthcare worker asked: | | |  |
| 85 | TLK_WT | If I knew how much weight I should gain during pregnancy | 0 | 0 |
| 86 | TLK_CIGS | If I was smoking cigarettes | 0 | 0 |
| 87 | TLK_ETOH | If I was drinking alcohol | 0 | 0 |
| 88 | ASK_MEDS | If I was taking any prescription medication | 0 | 0 |
| 89 | ASK_ABUS | If someone was hurting me emotionally or physically | 0 | 0 |
| 90 | ASK_DPRS | If I was feeling down or depressed | 0 | 0 |
| 91 | ASK_DRUG | If I was using drugs | 0 | 0 |
| 92 | ASK_HIVT | If I wanted to be tested for HIV | 0 | 0 |
| 93 | ASK_BF | If I planned to breastfeed my new baby | 0 | 0 |
| 94 | ASK_PPBC | If I planned to use birth control after my baby was born | 0 | 0 |
| 106 | BPG_DEPRS8 | Depression before pregnancy | 0 | 0 |
| 107 | MH_PGDX8 | Depression during pregnancy | 0 | 0 |
