# Supplementary material for: Race/ethnicity, disability, and antenatal depression in the United States: population-level insights from machine learning
Source: Prev Med Rep. 2026 Mar 7;65:103437. doi: 10.1016/j.pmedr.2026.103437 (PMC12996995; doi:10.1016/j.pmedr.2026.103437)
Supplement: Supplementary file 2 — Appendix B [file mmc2.docx]

| **Appendix B. Characteristics of Pregnant Women by Maternal Race/Ethnicity, 23 U.S. States and Jurisdictions, 2019 Pregnancy Risk Assessment Monitoring System (Full Version)** | | | | | | |
| --- | --- | --- | --- | --- | --- | --- |
|  | **Overall** N = 23,104^1^ | Race and Ethnicity | |  |  |  |
|  |  | **Non-Hispanic Black** N = 8,786^1^ | **Non-Hispanic White** N = 14,318^1^ | ***p*-value**^2^ | |  |
| State |  |  |  | <0.001 |  |  |
| Colorado | 942 (4.08%) | 64 (0.73%) | 878 (6.13%) |  |  |  |
| District of Columbia | 903 (3.91%) | 546 (6.21%) | 357 (2.49%) |  |  |  |
| Georgia | 1,031 (4.46%) | 608 (6.92%) | 423 (2.95%) |  |  |  |
| Kansas | 1,376 (5.96%) | 210 (2.39%) | 1,166 (8.14%) |  |  |  |
| Louisiana | 1,323 (5.73%) | 821 (9.34%) | 502 (3.51%) |  |  |  |
| Massachusetts | 990 (4.28%) | 649 (7.39%) | 341 (2.38%) |  |  |  |
| Maryland | 891 (3.86%) | 501 (5.70%) | 390 (2.72%) |  |  |  |
| Maine | 421 (1.82%) | 12 (0.14%) | 409 (2.86%) |  |  |  |
| Michigan | 3,026 (13.1%) | 1,662 (18.9%) | 1,364 (9.53%) |  |  |  |
| Missouri | 1,774 (7.68%) | 445 (5.06%) | 1,329 (9.28%) |  |  |  |
| Mississippi | 2,230 (9.65%) | 1,308 (14.9%) | 922 (6.44%) |  |  |  |
| Montana | 823 (3.56%) | 14 (0.16%) | 809 (5.65%) |  |  |  |
| North Dakota | 493 (2.13%) | 65 (0.74%) | 428 (2.99%) |  |  |  |
| Nebraska | 1,095 (4.74%) | 453 (5.16%) | 642 (4.48%) |  |  |  |
| New Hampshire | 185 (0.80%) | 2 (0.02%) | 183 (1.28%) |  |  |  |
| New Mexico | 948 (4.10%) | 39 (0.44%) | 909 (6.35%) |  |  |  |
| New York | 354 (1.53%) | 64 (0.73%) | 290 (2.03%) |  |  |  |
| Oregon | 1,609 (6.96%) | 571 (6.50%) | 1,038 (7.25%) |  |  |  |
| Rhode Island | 422 (1.83%) | 87 (0.99%) | 335 (2.34%) |  |  |  |
| South Dakota | 887 (3.84%) | 147 (1.67%) | 740 (5.17%) |  |  |  |
| Tennessee | 305 (1.32%) | 96 (1.09%) | 209 (1.46%) |  |  |  |
| Virginia | 955 (4.13%) | 417 (4.75%) | 538 (3.76%) |  |  |  |
| West Virginia | 121 (0.52%) | 5 (0.06%) | 116 (0.81%) |  |  |  |
| Rural Area |  |  |  | <0.001 |  |  |
| Rural | 6,010 (26.0%) | 1,189 (13.5%) | 4,821 (33.7%) |  |  |  |
| Urban | 17,073 (74.0%) | 7,589 (86.5%) | 9,484 (66.3%) |  |  |  |
| Unknown | 21 | 8 | 13 |  |  |  |
| Maternal Age | 28.3 (5.7) | 27.9 (5.9) | 28.5 (5.5) | <0.001 |  |  |
| Marital Status |  |  |  | <0.001 |  |  |
| Married | 11,010 (47.7%) | 2,285 (26.0%) | 8,725 (60.9%) |  |  |  |
| Other | 12,094 (52.3%) | 6,501 (74.0%) | 5,593 (39.1%) |  |  |  |
| Acknowledgement of Paternity |  |  |  | <0.001 |  |  |
| No | 15,050 (65.1%) | 4,919 (56.0%) | 10,131 (70.8%) |  |  |  |
| Yes | 8,054 (34.9%) | 3,867 (44.0%) | 4,187 (29.2%) |  |  |  |
| At Least One Disability |  |  |  | <0.001 |  |  |
| No | 12,588 (54.5%) | 5,119 (58.3%) | 7,469 (52.2%) |  |  |  |
| Yes | 10,516 (45.5%) | 3,667 (41.7%) | 6,849 (47.8%) |  |  |  |
| No. of Disabilities |  |  |  | <0.001 |  |  |
| 0 | 12,588 (54.5%) | 5,119 (58.3%) | 7,469 (52.2%) |  |  |  |
| 1 | 5,964 (25.8%) | 2,135 (24.3%) | 3,829 (26.7%) |  |  |  |
| 2 | 2,937 (12.7%) | 988 (11.2%) | 1,949 (13.6%) |  |  |  |
| 3 | 1,073 (4.6%) | 355 (4.0%) | 718 (5.0%) |  |  |  |
| 4 | 375 (1.6%) | 113 (1.3%) | 262 (1.8%) |  |  |  |
| 5 | 118 (0.5%) | 47 (0.5%) | 71 (0.5%) |  |  |  |
| 6 | 49 (0.2%) | 29 (0.3%) | 20 (0.1%) |  |  |  |
| Difficulty Seeing |  |  |  | 0.02 |  |  |
| No | 18,026 (78.0%) | 6,927 (78.8%) | 11,099 (77.5%) |  |  |  |
| Yes | 5,078 (22.0%) | 1,859 (21.2%) | 3,219 (22.5%) |  |  |  |
| Difficulty Seeing |  |  |  | <0.001 |  |  |
| 1 = No difficulty | 18,026 (78.0%) | 6,927 (78.8%) | 11,099 (77.5%) |  |  |  |
| 2 = Some difficulty | 4,514 (19.5%) | 1,564 (17.8%) | 2,950 (20.6%) |  |  |  |
| 3 = Lot of difficulty | 493 (2.1%) | 246 (2.8%) | 247 (1.7%) |  |  |  |
| 4 = Cannot do at all | 71 (0.3%) | 49 (0.6%) | 22 (0.2%) |  |  |  |
| Difficulty Hearing |  |  |  | <0.001 |  |  |
| No | 21,819 (94.4%) | 8,390 (95.5%) | 13,429 (93.8%) |  |  |  |
| Yes | 1,285 (5.6%) | 396 (4.5%) | 889 (6.2%) |  |  |  |
| Difficulty Hearing |  |  |  | <0.001 |  |  |
| 1 = No difficulty | 21,819 (94.4%) | 8,390 (95.5%) | 13,429 (93.8%) |  |  |  |
| 2 = Some difficulty | 1,104 (4 8%) | 316 (3.6%) | 788 (5.5%) |  |  |  |
| 3 = Lot of difficulty | 117 (0.5%) | 35 (0.4%) | 82 (0.6%) |  |  |  |
| 4 = Cannot do at all | 64 (0.3%) | 45 (0.5%) | 19 (0.1%) |  |  |  |
| Difficulty Walking |  |  |  | 0.5 |  |  |
| No | 21,398 (92.6%) | 8,123 (92.5%) | 13,275 (92.7%) |  |  |  |
| Yes | 1,706 (7.4%) | 663 (7.5%) | 1,043 (7.3%) |  |  |  |
| Difficulty Walking |  |  |  | <0.001 |  |  |
| 1 = No difficulty | 21,398 (92.6%) | 8,123 (92.5%) | 13,275 (92.7%) |  |  |  |
| 2 = Some difficulty | 1,470 (6.4%) | 542 (6.2%) | 928 (6.5%) |  |  |  |
| 3 = Lot of difficulty | 204 (0.9%) | 95 (1.1%) | 109 (0.8%) |  |  |  |
| 4 = Cannot do at all | 32 (0.1%) | 26 (0.3%) | 6 (0.0%) |  |  |  |
| Difficulty Remembering |  |  |  | <0.001 |  |  |
| No | 15,755 (68.2%) | 6,471 (73.7%) | 9,284 (64.8%) |  |  |  |
| Yes | 7,349 (31.8%) | 2,315 (26.3%) | 5,034 (35.2%) |  |  |  |
| Difficulty Remembering |  |  |  | <0.001 |  |  |
| 1 = No difficulty | 15,755 (68.2%) | 6,471 (73.7%) | 9,284 (64.8%) |  |  |  |
| 2 = Some difficulty | 6,178 (26.7%) | 1,940 (22.1%) | 4,238 (29.6%) |  |  |  |
| 3 = Lot of difficulty | 1,129 (4.9%) | 347 (3.9%) | 782 (5.5%) |  |  |  |
| 4 = Cannot do at all | 42 (0.2%) | 28 (0.3%) | 14 (0.1%) |  |  |  |
| Difficulty with Self-care |  |  |  | 0.007 |  |  |
| No | 22,377 (96.9%) | 8,544 (97.2%) | 13,833 (96.6%) |  |  |  |
| Yes | 727 (3.1%) | 242 (2.8%) | 485 (3.4%) |  |  |  |
| Difficulty with Self-care |  |  |  | <0.001 |  |  |
| 1 = No difficulty | 22,377 (96.9%) | 8,544 (97.2%) | 13,833 (96.6%) |  |  |  |
| 2 = Some difficulty | 633 (2.7%) | 198 (2.3%) | 435 (3.0%) |  |  |  |
| 3 = Lot of difficulty | 65 (0.3%) | 20 (0.2%) | 45 (0.3%) |  |  |  |
| 4 = Cannot do at all | 29 (0.1%) | 24 (0.3%) | 5 (0.0%) |  |  |  |
| Difficulty Communicating |  |  |  | <0.001 |  |  |
| No | 21,808 (94.4%) | 8,224 (93.6%) | 13,584 (94.9%) |  |  |  |
| Yes | 1,296 (5.6%) | 562 (6.4%) | 734 (5.1%) |  |  |  |
| Difficulty Communicating |  |  |  | <0.001 |  |  |
| 1 = No difficulty | 21,808 (94.4%) | 8,224 (93.6%) | 13,584 (94.9%) |  |  |  |
| 2 = Some difficulty | 1,119 (4.8%) | 468 (5.3%) | 651 (4.6%) |  |  |  |
| 3 = Lot of difficulty | 148 (0.6%) | 70 (0.8%) | 78 (0.5%) |  |  |  |
| 4 = Cannot do at all | 29 (0.1%) | 24 (0.3%) | 5 (0.0%) |  |  |  |
| Health Insurance Before Pregnancy |  |  |  | 0.7 |  |  |
| Insured | 20,090 (87.0%) | 7,626 (86.9%) | 12,464 (87.1%) |  |  |  |
| Uninsured | 2,996 (13.0%) | 1,149 (13.1%) | 1,847 (12.9%) |  |  |  |
| Unknown | 18 | 11 | 7 |  |  |  |
| Health Insurance During Pregnancy |  |  |  | <0.001 |  |  |
| Insured | 21,992 (95.2%) | 8,291 (94.4%) | 13,701 (95.7%) |  |  |  |
| Uninsured | 1,112 (4.8%) | 495 (5.6%) | 617 (4.3%) |  |  |  |
| Total Annual Income |  |  |  | <0.001 |  |  |
| 01. $0 to $16,000 | 5,378 (25.3%) | 2,955 (38.0%) | 2,423 (18.0%) |  |  |  |
| 02. $16,001 to $20,000 | 1,980 (9.3%) | 957 (12.3%) | 1,023 (7.6%) |  |  |  |
| 03. $20,001 to $24,000 | 1,518 (7.1%) | 658 (8.5%) | 860 (6.4%) |  |  |  |
| 04. $24,001 to $28,000 | 1,112 (5.2%) | 499 (6.4%) | 613 (4.6%) |  |  |  |
| 05. $28,001 to $32,000 | 1,377 (6.5%) | 514 (6.6%) | 863 (6.4%) |  |  |  |
| 06. $32,001 to $40,000 | 1,787 (8.4%) | 634 (8.2%) | 1,153 (8.6%) |  |  |  |
| 07. $40,001 to $48,000 | 1,376 (6.5%) | 406 (5.2%) | 970 (7.2%) |  |  |  |
| 08. $48,001 to $57,000 | 1,624 (7.6%) | 381 (4.9%) | 1,243 (9.2%) |  |  |  |
| 09. $57,001 to $60,000 | 1,017 (4.8%) | 200 (2.6%) | 817 (6.1%) |  |  |  |
| 10. $60,001 to $73,000 | 1,862 (8.8%) | 283 (3.6%) | 1,579 (11.7%) |  |  |  |
| 11. $73,001 to $85,000 | 1,804 (8.5%) | 227 (2.9%) | 1,577 (11.7%) |  |  |  |
| 12. $85,001 to $100,000 | 24 (0.1%) | 14 (0.2%) | 10 (0.1%) |  |  |  |
| 13. $100,001 to $120,000 | 34 (0.2%) | 11 (0.1%) | 23 (0.2%) |  |  |  |
| 14. $120,001+ | 331 (1.6%) | 34 (0.4%) | 297 (2.2%) |  |  |  |
| Unknown | 1,880 | 1,013 | 867 |  |  |  |
| No. of Household Members | 2.9 (1.5) | 2.8 (1.5) | 2.9 (1.4) | <0.001 |  |  |
| Unknown | 671 | 371 | 300 |  |  |  |
| Maternal Highest Degree |  |  |  | <0.001 |  |  |
| 1. < = 8th Grade | 291 (1.3%) | 106 (1.2%) | 185 (1.3%) |  |  |  |
| 2. 9-12th Grade | 2,088 (9.1%) | 1,008 (11.5%) | 1,080 (7.6%) |  |  |  |
| 3. High School Grad/GED | 7,022 (30.5%) | 3,199 (36.6%) | 3,823 (26.8%) |  |  |  |
| 4. Some College | 5,814 (25.3%) | 2,446 (28.0%) | 3,368 (23.6%) |  |  |  |
| 5. Associate Degree | 2,269 (9.9%) | 714 (8.2%) | 1,555 (10.9%) |  |  |  |
| 6. Bachelors Degree | 3,911 (17.0%) | 927 (10.6%) | 2,984 (20.9%) |  |  |  |
| 7. Masters Degree | 1,305 (5.7%) | 282 (3.2%) | 1,023 (7.2%) |  |  |  |
| 8. Doctorate/Professional Degree | 303 (1.3%) | 51 (0.6%) | 252 (1.8%) |  |  |  |
| Unknown | 101 | 53 | 48 |  |  |  |
| Special Supplemental Nutrition Program for Women, Infants, and Children During Pregnancy |  |  |  | <0.001 |  |  |
| No | 13,281 (57.5%) | 3,690 (42.0%) | 9,591 (67.0%) |  |  |  |
| Yes | 9,823 (42.5%) | 5,096 (58.0%) | 4,727 (33.0%) |  |  |  |
| Physical Abuse by Partner Before Pregnancy |  |  |  | <0.001 |  |  |
| No | 22,341 (96.7%) | 8,427 (95.9%) | 13,914 (97.2%) |  |  |  |
| Yes | 763 (3.3%) | 359 (4.1%) | 404 (2.8%) |  |  |  |
| Physical Abuse by Partner During Pregnancy |  |  |  | <0.001 |  |  |
| No | 22,430 (97.1%) | 8,458 (96.3%) | 13,972 (97.6%) |  |  |  |
| Yes | 674 (2.9%) | 328 (3.7%) | 346 (2.4%) |  |  |  |
| Physical Abuse by Ex-Partner Before Pregnancy |  |  |  | 0.01 |  |  |
| No | 22,211 (96.1%) | 8,410 (95.7%) | 13,801 (96.4%) |  |  |  |
| Yes | 893 (3.9%) | 376 (4.3%) | 517 (3.6%) |  |  |  |
| Physical Abuse by Ex-Partner During Pregnancy |  |  |  | <0.001 |  |  |
| No | 22,506 (97.4%) | 8,494 (96.7%) | 14,012 (97.9%) |  |  |  |
| Yes | 598 (2.6%) | 292 (3.3%) | 306 (2.1%) |  |  |  |
| No. of Loss of Pregnancy | 0.5 (1.0) | 0.6 (1.0) | 0.5 (0.9) | <0.001 |  |  |
| Unknown | 39 | 18 | 21 |  |  |  |
| Infertility Treatment |  |  |  | <0.001 |  |  |
| No | 22,822 (98.9%) | 8,729 (99.4%) | 14,093 (98.5%) |  |  |  |
| Yes | 265 (1.1%) | 50 (0.6%) | 215 (1.5%) |  |  |  |
| Unknown | 17 | 7 | 10 |  |  |  |
| Diabetes Before Pregnancy |  |  |  | <0.001 |  |  |
| No | 22,166 (95.9%) | 8,375 (95.3%) | 13,791 (96.3%) |  |  |  |
| Yes | 938 (4.1%) | 411 (4.7%) | 527 (3.7%) |  |  |  |
| Hypertension Before Pregnancy |  |  |  | <0.001 |  |  |
| No | 21,226 (91.9%) | 7,799 (88.8%) | 13,427 (93.8%) |  |  |  |
| Yes | 1,878 (8.1%) | 987 (11.2%) | 891 (6.2%) |  |  |  |
| Diabetes During Pregnancy |  |  |  | 0.2 |  |  |
| No | 20,658 (89.4%) | 7,883 (89.7%) | 12,775 (89.2%) |  |  |  |
| Yes | 2,446 (10.6%) | 903 (10.3%) | 1,543 (10.8%) |  |  |  |
| Hypertension During Pregnancy |  |  |  | <0.001 |  |  |
| No | 18,357 (79.5%) | 6,766 (77.0%) | 11,591 (81.0%) |  |  |  |
| Yes | 4,747 (20.5%) | 2,020 (23.0%) | 2,727 (19.0%) |  |  |  |
| Body Mass Index Before Pregnancy | 28.0 (7.6) | 29.1 (8.0) | 27.4 (7.2) | <0.001 |  |  |
| Unknown | 642 | 353 | 289 |  |  |  |
| Maternal Weight Gain (Lbs) | 28.7 (16.3) | 27.1 (16.9) | 29.7 (15.8) | <0.001 |  |  |
| Unknown | 463 | 223 | 240 |  |  |  |
| Pregnancy Intention |  |  |  | <0.001 |  |  |
| Later | 4,808 (21.1%) | 2,229 (25.7%) | 2,579 (18.3%) |  |  |  |
| Not sure | 4,630 (20.3%) | 2,179 (25.1%) | 2,451 (17.4%) |  |  |  |
| Not want | 1,902 (8.3%) | 971 (11.2%) | 931 (6.6%) |  |  |  |
| Sooner | 2,736 (12.0%) | 714 (8.2%) | 2,022 (14.3%) |  |  |  |
| Then | 8,705 (38.2%) | 2,580 (29.7%) | 6,125 (43.4%) |  |  |  |
| Unknown | 323 | 113 | 210 |  |  |  |
| No. Cigarettes Before Pregnancy | 1.7 (5.8) | 0.9 (4.2) | 2.3 (6.5) | <0.001 |  |  |
| Unknown | 99 | 50 | 49 |  |  |  |
| No. Cigarettes in 1st Trimester | 1.1 (4.3) | 0.6 (3.2) | 1.5 (4.9) | <0.001 |  |  |
| Unknown | 100 | 51 | 49 |  |  |  |
| No. Cigarettes in 2nd Trimester | 0.9 (3.6) | 0.4 (2.7) | 1.1 (4.0) | <0.001 |  |  |
| Unknown | 96 | 49 | 47 |  |  |  |
| No. Cigarettes in 3rd Trimester | 0.8 (3.5) | 0.4 (2.6) | 1.0 (4.0) | <0.001 |  |  |
| Unknown | 97 | 50 | 47 |  |  |  |
| E-Cigarettes Before Pregnancy |  |  |  | <0.001 |  |  |
| 1. Not use | 21,402 (93.4%) | 8,438 (96.7%) | 12,964 (91.3%) |  |  |  |
| 2. 1 day a week or less | 456 (2.0%) | 102 (1.2%) | 354 (2.5%) |  |  |  |
| 3. 2-6 days a week | 209 (0.9%) | 52 (0.6%) | 157 (1.1%) |  |  |  |
| 4. Once a day | 163 (0.7%) | 40 (0.5%) | 123 (0.9%) |  |  |  |
| 5. More than once a day | 696 (3.0%) | 93 (1.1%) | 603 (4.2%) |  |  |  |
| Unknown | 178 | 61 | 117 |  |  |  |
| E-Cigarettes During Pregnancy |  |  |  | <0.001 |  |  |
| 1. Not use | 22,481 (98.0%) | 8,661 (99.2%) | 13,820 (97.2%) |  |  |  |
| 2. 1 day a week or less | 153 (0.7%) | 28 (0.3%) | 125 (0.9%) |  |  |  |
| 3. 2-6 days a week | 85 (0.4%) | 15 (0.2%) | 70 (0.5%) |  |  |  |
| 4. Once a day | 62 (0.3%) | 13 (0.1%) | 49 (0.3%) |  |  |  |
| 5. More than once a day | 169 (0.7%) | 16 (0.2%) | 153 (1.1%) |  |  |  |
| Unknown | 154 | 53 | 101 |  |  |  |
| Drinking in the Last 2 Years |  |  |  | <0.001 |  |  |
| No | 7,735 (33.5%) | 3,966 (45.1%) | 3,769 (26.3%) |  |  |  |
| Yes | 15,369 (66.5%) | 4,820 (54.9%) | 10,549 (73.7%) |  |  |  |
| Pre-pregnancy Healthcare Visit |  |  |  | <0.001 |  |  |
| No | 8,061 (34.9%) | 3,678 (41.9%) | 4,383 (30.6%) |  |  |  |
| Yes | 15,043 (65.1%) | 5,108 (58.1%) | 9,935 (69.4%) |  |  |  |
| Pre-pregnancy Checkup with Doctor |  |  |  | <0.001 |  |  |
| No | 6,721 (29.1%) | 1,716 (19.5%) | 5,005 (35.0%) |  |  |  |
| Yes | 16,383 (70.9%) | 7,070 (80.5%) | 9,313 (65.0%) |  |  |  |
| Pre-pregnancy Checkup with Obstetrician–Gynecologist |  |  |  | <0.001 |  |  |
| No | 6,564 (28.4%) | 1,923 (21.9%) | 4,641 (32.4%) |  |  |  |
| Yes | 16,540 (71.6%) | 6,863 (78.1%) | 9,677 (67.6%) |  |  |  |
| Pre-pregnancy Visit for Illness |  |  |  | <0.001 |  |  |
| No | 11,977 (51.8%) | 4,274 (48.6%) | 7,703 (53.8%) |  |  |  |
| Yes | 11,127 (48.2%) | 4,512 (51.4%) | 6,615 (46.2%) |  |  |  |
| Pre-pregnancy Visit for Injury |  |  |  | <0.001 |  |  |
| No | 13,896 (60.1%) | 4,715 (53.7%) | 9,181 (64.1%) |  |  |  |
| Yes | 9,208 (39.9%) | 4,071 (46.3%) | 5,137 (35.9%) |  |  |  |
| Pre-pregnancy Visit for Family Planning/Birth Control |  |  |  | <0.001 |  |  |
| No | 12,040 (52.1%) | 4,091 (46.6%) | 7,949 (55.5%) |  |  |  |
| Yes | 11,064 (47.9%) | 4,695 (53.4%) | 6,369 (44.5%) |  |  |  |
| Pre-pregnancy Visit for Depression or Anxiety |  |  |  | <0.001 |  |  |
| No | 12,154 (52.6%) | 4,367 (49.7%) | 7,787 (54.4%) |  |  |  |
| Yes | 10,950 (47.4%) | 4,419 (50.3%) | 6,531 (45.6%) |  |  |  |
| Pre-pregnancy Visit with Dentist |  |  |  | <0.001 |  |  |
| No | 6,501 (28.1%) | 2,357 (26.8%) | 4,144 (28.9%) |  |  |  |
| Yes | 16,603 (71.9%) | 6,429 (73.2%) | 10,174 (71.1%) |  |  |  |
| Pre-pregnancy Other Healthcare |  |  |  | <0.001 |  |  |
| No | 12,836 (55.6%) | 4,563 (51.9%) | 8,273 (57.8%) |  |  |  |
| Yes | 10,268 (44.4%) | 4,223 (48.1%) | 6,045 (42.2%) |  |  |  |
| During pre-pregnancy healthcare visits, a healthcare worker did: | | | | |  |  |
| Tell me to take a vitamin with folic acid |  |  |  | <0.001 |  |  |
| No | 10,465 (45.3%) | 3,745 (42.6%) | 6,720 (46.9%) |  |  |  |
| Yes | 12,639 (54.7%) | 5,041 (57.4%) | 7,598 (53.1%) |  |  |  |
| Talk to me about maintaining a healthy weight |  |  |  | <0.001 |  |  |
| No | 8,852 (38.3%) | 2,565 (29.2%) | 6,287 (43.9%) |  |  |  |
| Yes | 14,252 (61.7%) | 6,221 (70.8%) | 8,031 (56.1%) |  |  |  |
| Talk to me about controlling any medical conditions such as diabetes or high blood pressure |  |  |  | <0.001 |  |  |
| No | 12,119 (52.5%) | 3,635 (41.4%) | 8,484 (59.3%) |  |  |  |
| Yes | 10,985 (47.5%) | 5,151 (58.6%) | 5,834 (40.7%) |  |  |  |
| Talk to me about my desire to have or not have children |  |  |  | <0.001 |  |  |
| No | 8,821 (38.2%) | 3,162 (36.0%) | 5,659 (39.5%) |  |  |  |
| Yes | 14,283 (61.8%) | 5,624 (64.0%) | 8,659 (60.5%) |  |  |  |
| Talk to me about using birth control to prevent pregnancy |  |  |  | <0.001 |  |  |
| No | 7,837 (33.9%) | 2,165 (24.6%) | 5,672 (39.6%) |  |  |  |
| Yes | 15,267 (66.1%) | 6,621 (75.4%) | 8,646 (60.4%) |  |  |  |
| Talk to me about how I could improve my health before a pregnancy |  |  |  | <0.001 |  |  |
| No | 10,237 (44.3%) | 3,199 (36.4%) | 7,038 (49.2%) |  |  |  |
| Yes | 12,867 (55.7%) | 5,587 (63.6%) | 7,280 (50.8%) |  |  |  |
| Talk to me about sexually transmitted infections |  |  |  | <0.001 |  |  |
| No | 9,837 (42.6%) | 2,494 (28.4%) | 7,343 (51.3%) |  |  |  |
| Yes | 13,267 (57.4%) | 6,292 (71.6%) | 6,975 (48.7%) |  |  |  |
| Ask me if I was smoking cigarettes |  |  |  | <0.001 |  |  |
| No | 3,031 (13.1%) | 967 (11.0%) | 2,064 (14.4%) |  |  |  |
| Yes | 20,073 (86.9%) | 7,819 (89.0%) | 12,254 (85.6%) |  |  |  |
| Ask me if someone was hurting me emotionally or physically |  |  |  | <0.001 |  |  |
| No | 6,149 (26.6%) | 1,793 (20.4%) | 4,356 (30.4%) |  |  |  |
| Yes | 16,955 (73.4%) | 6,993 (79.6%) | 9,962 (69.6%) |  |  |  |
| Ask me if I was feeling down or depressed |  |  |  | <0.001 |  |  |
| No | 5,262 (22.8%) | 1,599 (18.2%) | 3,663 (25.6%) |  |  |  |
| Yes | 17,842 (77.2%) | 7,187 (81.8%) | 10,655 (74.4%) |  |  |  |
| Ask me about the kind of work I do |  |  |  | <0.001 |  |  |
| No | 5,108 (22.1%) | 1,665 (19.0%) | 3,443 (24.0%) |  |  |  |
| Yes | 17,996 (77.9%) | 7,121 (81.0%) | 10,875 (76.0%) |  |  |  |
| Test me for Human Immunodeficiency Virus (HIV) |  |  |  | <0.001 |  |  |
| No | 9,299 (40.2%) | 1,991 (22.7%) | 7,308 (51.0%) |  |  |  |
| Yes | 13,805 (59.8%) | 6,795 (77.3%) | 7,010 (49.0%) |  |  |  |
| Start of Prenatal Care in 1st Trimester |  |  |  | <0.001 |  |  |
| No | 2,946 (13.1%) | 1,485 (17.5%) | 1,461 (10.4%) |  |  |  |
| No prenatal care | 246 (1.1%) | 127 (1.5%) | 119 (0.8%) |  |  |  |
| Yes | 19,323 (85.8%) | 6,852 (81.0%) | 12,471 (88.8%) |  |  |  |
| Unknown | 589 | 322 | 267 |  |  |  |
| No. of Prenatal Care Visits | 11.1 (4.5) | 10.6 (4.9) | 11.3 (4.2) | <0.001 |  |  |
| Unknown | 493 | 237 | 256 |  |  |  |
| Kessner Index |  |  |  | <0.001 |  |  |
| 1. Unknown | 1,043 (4.5%) | 461 (5.2%) | 582 (4.0%) |  |  |  |
| 2. Inadequate | 1,597 (6.9%) | 814 (9.3%) | 783 (5.5%) |  |  |  |
| 3. Intermediate | 4,762 (20.6%) | 2,154 (24.5%) | 2,608 (18.2%) |  |  |  |
| 4. Adequate | 15,702 (68.0%) | 5,357 (61.0%) | 10,345 (72.3%) |  |  |  |
| Kotelchuck Index |  |  |  | <0.001 |  |  |
| 1. Inadequate | 2,991 (13.2%) | 1,443 (16.9%) | 1,548 (11.0%) |  |  |  |
| 2. Intermediate | 2,245 (9.92%) | 955 (11.2%) | 1,290 (9.17%) |  |  |  |
| 3. Adequate | 9,187 (40.6%) | 2,916 (34.1%) | 6,271 (44.6%) |  |  |  |
| 4. Adequate plus | 8,201 (36.2%) | 3,236 (37.8%) | 4,965 (35.3%) |  |  |  |
| Unknown | 480 | 236 | 244 |  |  |  |
| During Prenatal Care visits, a healthcare worker asked: | | | | |  |  |
| Ask if I knew how much weight I should gain during pregnancy |  |  |  | <0.001 |  |  |
| No | 10,033 (43.4%) | 3,539 (40.3%) | 6,494 (45.4%) |  |  |  |
| Yes | 13,071 (56.6%) | 5,247 (59.7%) | 7,824 (54.6%) |  |  |  |
| Ask if I was smoking cigarettes |  |  |  | <0.001 |  |  |
| No | 883 (3.8%) | 427 (4.9%) | 456 (3.2%) |  |  |  |
| Yes | 22,221 (96.2%) | 8,359 (95.1%) | 13,862 (96.8%) |  |  |  |
| Ask if I was drinking alcohol |  |  |  | <0.001 |  |  |
| No | 1,062 (4.6%) | 479 (5.5%) | 583 (4.1%) |  |  |  |
| Yes | 22,042 (95.4%) | 8,307 (94.5%) | 13,735 (95.9%) |  |  |  |
| Ask if I was taking any prescription medication |  |  |  | <0.001 |  |  |
| No | 1,011 (4.4%) | 580 (6.6%) | 431 (3.0%) |  |  |  |
| Yes | 22,093 (95.6%) | 8,206 (93.4%) | 13,887 (97.0%) |  |  |  |
| Ask if someone was hurting me emotionally or physically |  |  |  | <0.001 |  |  |
| No | 4,771 (20.7%) | 1,474 (16.8%) | 3,297 (23.0%) |  |  |  |
| Yes | 18,333 (79.3%) | 7,312 (83.2%) | 11,021 (77.0%) |  |  |  |
| Ask if I was feeling down or depressed |  |  |  | <0.001 |  |  |
| No | 3,474 (15.0%) | 1,071 (12.2%) | 2,403 (16.8%) |  |  |  |
| Yes | 19,630 (85.0%) | 7,715 (87.8%) | 11,915 (83.2%) |  |  |  |
| Ask if I was using drugs such as marijuana, cocaine, crack, or meth |  |  |  | <0.001 |  |  |
| No | 3,230 (14.0%) | 1,017 (11.6%) | 2,213 (15.5%) |  |  |  |
| Yes | 19,874 (86.0%) | 7,769 (88.4%) | 12,105 (84.5%) |  |  |  |
| Ask if I wanted to be tested for HIV |  |  |  | <0.001 |  |  |
| No | 8,441 (36.5%) | 2,379 (27.1%) | 6,062 (42.3%) |  |  |  |
| Yes | 14,663 (63.5%) | 6,407 (72.9%) | 8,256 (57.7%) |  |  |  |
| Ask if I planned to breastfeed my new baby |  |  |  | <0.001 |  |  |
| No | 1,622 (7.0%) | 438 (5.0%) | 1,184 (8.3%) |  |  |  |
| Yes | 21,482 (93.0%) | 8,348 (95.0%) | 13,134 (91.7%) |  |  |  |
| Ask if I planned to use birth control after my baby was born |  |  |  | <0.001 |  |  |
| No | 3,274 (14.2%) | 759 (8.6%) | 2,515 (17.6%) |  |  |  |
| Yes | 19,830 (85.8%) | 8,027 (91.4%) | 11,803 (82.4%) |  |  |  |
| Depression Before Pregnancy |  |  |  | <0.001 |  |  |
| No | 17,904 (77.5%) | 7,197 (81.9%) | 10,707 (74.8%) |  |  |  |
| Yes | 5,200 (22.5%) | 1,589 (18.1%) | 3,611 (25.2%) |  |  |  |
| Depression During Pregnancy |  |  |  | <0.001 |  |  |
| No | 17,910 (77.5%) | 6,961 (79.2%) | 10,949 (76.5%) |  |  |  |
| Yes | 5,194 (22.5%) | 1,825 (20.8%) | 3,369 (23.5%) |  |  |  |
| ^1^n (%); Mean (SD)  ^2^Pearson’s Chi-squared test; Wilcoxon rank sum test | | | | | | |
