# Supplementary material for: Race/ethnicity, disability, and antenatal depression in the United States: population-level insights from machine learning
Source: Prev Med Rep. 2026 Mar 7;65:103437. doi: 10.1016/j.pmedr.2026.103437 (PMC12996995; doi:10.1016/j.pmedr.2026.103437)
Supplement: Supplementary file 3 — Appendix C [file mmc3.docx]

Appendix C. Overall Association with Antenatal Depression Among Pregnant Women in 23 U.S. States and Jurisdictions, 2019 Pregnancy Risk Assessment Monitoring System

| **Characteristic** | **Overall** N = 23,104^1^ | **No** N = 17,910^1^ | **Yes** N = 5,194^1^ | ***p*-value^2^** |
| --- | --- | --- | --- | --- |
| Race/Ethnicity |  |  |  | <0.001 |
| Non-Hispanic Black | 8,786 (100.0%) | 6,961 (79.2%) | 1,825 (20.8%) |  |
| Non-Hispanic White | 14,318 (100.0%) | 10,949 (76.5%) | 3,369 (23.5%) |  |
| Maternal Age | 28.3 (5.7) | 28.5 (5.6) | 27.4 (5.7) | <0.001 |
| Marital Status |  |  |  | <0.001 |
| Married | 11,010 (100.0%) | 9,117 (82.8%) | 1,893 (17.2%) |  |
| Other | 12,094 (100.0%) | 8,793 (72.7%) | 3,301 (27.3%) |  |
| Acknowledgement of Paternity |  |  |  | <0.001 |
| No | 15,050 (100.0%) | 11,967 (79.5%) | 3,083 (20.5%) |  |
| Yes | 8,054 (100.0%) | 5,943 (73.8%) | 2,111 (26.2%) |  |
| Rural Area |  |  |  | 0.3 |
| Rural | 6,010 (100.0%) | 4,631 (77.1%) | 1,379 (22.9%) |  |
| Urban | 17,073 (100.0%) | 13,264 (77.7%) | 3,809 (22.3%) |  |
| Unknown | 21 | 15 | 6 |  |
| At Least One Disability |  |  |  | <0.001 |
| No | 12,588 (100.0%) | 10,951 (87.0%) | 1,637 (13.0%) |  |
| Yes | 10,516 (100.0%) | 6,959 (66.2%) | 3,557 (33.8%) |  |
| No. of Disabilities |  |  |  | <0.001 |
| 0 | 12,588 (100.0%) | 10,951 (87.0%) | 1,637 (13.0%) |  |
| 1 | 5,964 (100.0%) | 4,379 (73.4%) | 1,585 (26.6%) |  |
| 2 | 2,937 (100.0%) | 1,792 (61.0%) | 1,145 (39.0%) |  |
| 3 | 1,073 (100.0%) | 574 (53.5%) | 499 (46.5%) |  |
| 4 | 375 (100.0%) | 153 (40.8%) | 222 (59.2%) |  |
| 5 | 118 (100.0%) | 37 (31.4%) | 81 (68.6%) |  |
| 6 | 49.0 (100.0%) | 24 (49.0%) | 25 (51.0%) |  |
| Difficulty Seeing |  |  |  | <0.001 |
| No | 18,026 (100.0%) | 14,604 (81.0%) | 3,422 (19.0%) |  |
| Yes | 5,078 (100.0%) | 3,306 (65.1%) | 1,772 (34.9%) |  |
| Difficulty Seeing |  |  |  | <0.001 |
| 1 = No difficulty | 18,026 (100.0%) | 14,604 (81.0%) | 3,422 (19.0%) |  |
| 2 = Some difficulty | 4,514 (100.0%) | 2,962 (65.6%) | 1,552 (34.4%) |  |
| 3 = Lot of difficulty | 493 (100.0%) | 293 (59.4%) | 200 (40.6%) |  |
| 4 = Cannot do at all | 71 (100.0%) | 51 (71.8%) | 20 (28.2%) |  |
| Difficulty Hearing |  |  |  | <0.001 |
| No | 21,819 (100.0%) | 17,143 (78.6%) | 4,676 (21.4%) |  |
| Yes | 1,285 (100.0%) | 767 (59.7%) | 518 (40.3%) |  |
| Difficulty Hearing |  |  |  | <0.001 |
| 1 = No difficulty | 21,819 (100.0%) | 17,143 (78.6%) | 4,676 (21.4%) |  |
| 2 = Some difficulty | 1,104 (100.0%) | 664 (60.1%) | 440 (39.9%) |  |
| 3 = Lot of difficulty | 117 (100.0%) | 60 (51.3%) | 57 (48.7%) |  |
| 4 = Cannot do at all | 64 (100.0%) | 43 (67.2%) | 21 (32.8%) |  |
| Difficulty Walking |  |  |  | <0.001 |
| No | 21,398 (100.0%) | 16,937 (79.2%) | 4,461 (20.8%) |  |
| Yes | 1,706 (100.0%) | 973 (57.0%) | 733 (43.0%) |  |
| Difficulty Walking |  |  |  | <0.001 |
| 1 = No difficulty | 21,398 (100.0%) | 16,937 (79.2%) | 4,461 (20.8%) |  |
| 2 = Some difficulty | 1,470 (100.0%) | 845 (57.5%) | 625 (42.5%) |  |
| 3 = Lot of difficulty | 204 (100.0%) | 106 (52.0%) | 98 (48.0%) |  |
| 4 = Cannot do at all | 32 (100.0%) | 22 (68.8%) | 10 (31.3%) |  |
| Difficulty Remembering |  |  |  | <0.001 |
| No | 15,755 (100.0%) | 13,388 (85.0%) | 2,367 (15.0%) |  |
| Yes | 7,349 (100.0%) | 4,522 (61.5%) | 2,827 (38.5%) |  |
| Difficulty Remembering |  |  |  | <0.001 |
| 1 = No difficulty | 15,755 (100.0%) | 13,388 (85.0%) | 2,367 (15.0%) |  |
| 2 = Some difficulty | 6,178 (100.0%) | 4,010 (64.9%) | 2,168 (35.1%) |  |
| 3 = Lot of difficulty | 1,129 (100.0%) | 488 (43.2%) | 641 (56.8%) |  |
| 4 = Cannot do at all | 42 (100.0%) | 24 (57.1%) | 18 (42.9%) |  |
| Difficulty with Self-care |  |  |  | <0.001 |
| No | 22,377 (100.0%) | 17,582 (78.6%) | 4,795 (21.4%) |  |
| Yes | 727 (100.0%) | 328 (45.1%) | 399 (54.9%) |  |
| Difficulty with Self-care |  |  |  | <0.001 |
| 1 = No difficulty | 22,377 (100.0%) | 17,582 (78.6%) | 4,795 (21.4%) |  |
| 2 = Some difficulty | 633 (100.0%) | 288 (45.5%) | 345 (54.5%) |  |
| 3 = Lot of difficulty | 65 (100.0%) | 23 (35.4%) | 42 (64.6%) |  |
| 4 = Cannot do at all | 29 (100.0%) | 17 (58.6%) | 12 (41.4%) |  |
| Difficulty Communicating |  |  |  | <0.001 |
| No | 21,808 (100.0%) | 17,180 (78.8%) | 4,628 (21.2%) |  |
| Yes | 1,296 (100.0%) | 730 (56.3%) | 566 (43.7%) |  |
| Difficulty Communicating |  |  |  | <0.001 |
| 1 = No difficulty | 21,808 (100.0%) | 17,180 (78.8%) | 4,628 (21.2%) |  |
| 2 = Some difficulty | 1,119 (100.0%) | 646 (57.7%) | 473 (42.3%) |  |
| 3 = Lot of difficulty | 148 (100.0%) | 66 (44.6%) | 82 (55.4%) |  |
| 4 = Cannot do at all | 29 (100.0%) | 18 (62.1%) | 11 (37.9%) |  |
| Health Insurance Before Pregnancy |  |  |  | 0.02 |
| Insured | 20,090 (100.0%) | 15,625 (77.8%) | 4,465 (22.2%) |  |
| Uninsured | 2,996 (100.0%) | 2,273 (75.9%) | 723 (24.1%) |  |
| Unknown | 18 | 12 | 6 |  |
| Health Insurance During Pregnancy |  |  |  | 0.11 |
| Insured | 21,992 (100.0%) | 17,070 (77.6%) | 4,922 (22.4%) |  |
| Uninsured | 1,112 (100.0%) | 840 (75.5%) | 272 (24.5%) |  |
| Total Annual Income |  |  |  | <0.001 |
| 01. $0 to $16,000 | 5,378 (100.0%) | 3,690 (68.6%) | 1,688 (31.4%) |  |
| 02. $16,001 to $20,000 | 1,980 (100.0%) | 1,460 (73.7%) | 520 (26.3%) |  |
| 03. $20,001 to $24,000 | 1,518 (100.0%) | 1,144 (75.4%) | 374 (24.6%) |  |
| 04. $24,001 to $28,000 | 1,112 (100.0%) | 867 (78.0%) | 245 (22.0%) |  |
| 05. $28,001 to $32,000 | 1,377 (100.0%) | 1,071 (77.8%) | 306 (22.2%) |  |
| 06. $32,001 to $40,000 | 1,787 (100.0%) | 1,405 (78.6%) | 382 (21.4%) |  |
| 07. $40,001 to $48,000 | 1,376 (100.0%) | 1,112 (80.8%) | 264 (19.2%) |  |
| 08. $48,001 to $57,000 | 1,624 (100.0%) | 1,319 (81.2%) | 305 (18.8%) |  |
| 09. $57,001 to $60,000 | 1,017 (100.0%) | 828 (81.4%) | 189 (18.6%) |  |
| 10. $60,001 to $73,000 | 1,862 (100.0%) | 1,599 (85.9%) | 263 (14.1%) |  |
| 11. $73,001 to $85,000 | 1,804 (100.0%) | 1,542 (85.5%) | 262 (14.5%) |  |
| 12. $85,001 to $100,000 | 24 (100.0%) | 19 (79.2%) | 5 (20.8%) |  |
| 13. $100,001 to $120,000 | 34 (100.0%) | 31 (91.2%) | 3 (8.82%) |  |
| 14. $120,001+ | 331 (100.0%) | 312 (94.3%) | 19 (5.74%) |  |
| Unknown | 1,880 | 1,511 | 369 |  |
| No. of Household Members | 2.9 (1.5) | 2.9 (1.4) | 2.9 (1.5) | 0.7 |
| Unknown | 671 | 546 | 125 |  |
| Maternal Highest Degree |  |  |  | <0.001 |
| 1. < = 8th Grade | 291 (100.0%) | 239 (82.1%) | 52 (17.9%) |  |
| 2. 9-12th Grade | 2,088 (100.0%) | 1,410 (67.5%) | 678 (32.5%) |  |
| 3. High School Grad/GED | 7,022 (100.0%) | 5,211 (74.2%) | 1,811 (25.8%) |  |
| 4. Some College | 5,814 (100.0%) | 4,360 (75.0%) | 1,454 (25.0%) |  |
| 5. Associate Degree | 2,269 (100.0%) | 1,824 (80.4%) | 445 (19.6%) |  |
| 6. Bachelors Degree | 3,911 (100.0%) | 3,396 (86.8%) | 515 (13.2%) |  |
| 7. Masters Degree | 1,305 (100.0%) | 1,125 (86.2%) | 180 (13.8%) |  |
| 8. Doctorate/Professional Degree | 303 (100.0%) | 268 (88.4%) | 35 (11.6%) |  |
| Unknown | 101 | 77 | 24 |  |
| Special Supplemental Nutrition Program for Women, Infants, and Children During Pregnancy |  |  |  | <0.001 |
| No | 13,281 (100.0%) | 10,727 (80.8%) | 2,554 (19.2%) |  |
| Yes | 9,823 (100.0%) | 7,183 (73.1%) | 2,640 (26.9%) |  |
| Physical Abuse by Partner Before Pregnancy |  |  |  | <0.001 |
| No | 22,341 (100.0%) | 17,538 (78.5%) | 4,803 (21.5%) |  |
| Yes | 763 (100.0%) | 372 (48.8%) | 391 (51.2%) |  |
| Physical Abuse by Partner During Pregnancy |  |  |  | <0.001 |
| No | 22,430 (100.0%) | 17,579 (78.4%) | 4,851 (21.6%) |  |
| Yes | 674 (100.0%) | 331 (49.1%) | 343 (50.9%) |  |
| Physical Abuse by Ex-Partner Before Pregnancy |  |  |  | <0.001 |
| No | 22,211 (100.0%) | 17,458 (78.6%) | 4,753 (21.4%) |  |
| Yes | 893 (100.0%) | 452 (50.6%) | 441 (49.4%) |  |
| Physical Abuse by Ex-Partner During Pregnancy |  |  |  | <0.001 |
| No | 22,506 (100.0%) | 17,599 (78.2%) | 4,907 (21.8%) |  |
| Yes | 598 (100.0%) | 311 (52.0%) | 287 (48.0%) |  |
| No. of Loss of Pregnancy | 0.5 (1.0) | 0.5 (0.9) | 0.6 (1.1) | <0.001 |
| Unknown | 39 | 32 | 7 |  |
| Infertility Treatment |  |  |  | <0.001 |
| No | 22,822 (100.0%) | 17,666 (77.4%) | 5,156 (22.6%) |  |
| Yes | 265 (100.0%) | 233 (87.9%) | 32 (12.1%) |  |
| Unknown | 17 | 11 | 6 |  |
| Diabetes Before Pregnancy |  |  |  | <0.001 |
| No | 22,166 (100.0%) | 17,243 (77.8%) | 4,923 (22.2%) |  |
| Yes | 938 (100.0%) | 667 (71.1%) | 271 (28.9%) |  |
| Hypertension Before Pregnancy |  |  |  | <0.001 |
| No | 21,226 (100.0%) | 16,647 (78.4%) | 4,579 (21.6%) |  |
| Yes | 1,878 (100.0%) | 1,263 (67.3%) | 615 (32.7%) |  |
| Diabetes During Pregnancy |  |  |  | <0.001 |
| No | 20,658 (100.0%) | 16,327 (79.0%) | 4,331 (21.0%) |  |
| Yes | 2,446 (100.0%) | 1,583 (64.7%) | 863 (35.3%) |  |
| Hypertension During Pregnancy |  |  |  | <0.001 |
| No | 18,357 (100.0%) | 14,798 (80.6%) | 3,559 (19.4%) |  |
| Yes | 4,747 (100.0%) | 3,112 (65.6%) | 1,635 (34.4%) |  |
| Body Mass Index Before Pregnancy | 28.0 (7.6) | 27.8 (7.4) | 28.9 (8.2) | <0.001 |
| Unknown | 642 | 497 | 145 |  |
| Maternal Weight Gain (lbs) | 28.7 (16.3) | 28.9 (15.9) | 28.1 (17.3) | <0.001 |
| Unknown | 463 | 347 | 116 |  |
| Pregnancy Intention |  |  |  | <0.001 |
| Later | 4,808 (100.0%) | 3,607 (75.0%) | 1,201 (25.0%) |  |
| Not sure | 4,630 (100.0%) | 3,326 (71.8%) | 1,304 (28.2%) |  |
| Not want | 1,902 (100.0%) | 1,221 (64.2%) | 681 (35.8%) |  |
| Sooner | 2,736 (100.0%) | 2,209 (80.7%) | 527 (19.3%) |  |
| Then | 8,705 (100.0%) | 7,326 (84.2%) | 1,379 (15.8%) |  |
| Unknown | 323 | 221 | 102 |  |
| No. Cigarettes Before Pregnancy | 1.7 (5.8) | 1.4 (5.1) | 3.1 (7.4) | <0.001 |
| Unknown | 99 | 67 | 32 |  |
| No. Cigarettes in 1st Trimester | 1.1 (4.3) | 0.9 (3.8) | 2.1 (5.8) | <0.001 |
| Unknown | 100 | 67 | 33 |  |
| No. Cigarettes in 2nd Trimester | 0.9 (3.6) | 0.6 (3.1) | 1.6 (4.9) | <0.001 |
| Unknown | 96 | 66 | 30 |  |
| No. Cigarettes in 3rd Trimester | 0.8 (3.5) | 0.6 (3.0) | 1.4 (5.0) | <0.001 |
| Unknown | 97 | 66 | 31 |  |
| E-Cigarettes Before Pregnancy |  |  |  | <0.001 |
| 1. Not use | 21,402 (100.0%) | 16,947 (79.2%) | 4,455 (20.8%) |  |
| 2. 1 day a week or less | 456 (100.0%) | 239 (52.4%) | 217 (47.6%) |  |
| 3. 2-6 days a week | 209 (100.0%) | 125 (59.8%) | 84 (40.2%) |  |
| 4. Once a day | 163 (100.0%) | 100 (61.3%) | 63 (38.7%) |  |
| 5. More than once a day | 696 (100.0%) | 405 (58.2%) | 291 (41.8%) |  |
| Unknown | 178 | 94 | 84 |  |
| E-Cigarettes During Pregnancy |  |  |  | <0.001 |
| 1. Not use | 22,481 (100.0%) | 17,579 (78.2%) | 4,902 (21.8%) |  |
| 2. 1 day a week or less | 153 (100.0%) | 70 (45.8%) | 83 (54.2%) |  |
| 3. 2-6 days a week | 85 (100.0%) | 42 (49.4%) | 43 (50.6%) |  |
| 4. Once a day | 62 (100.0%) | 32 (51.6%) | 30 (48.4%) |  |
| 5. More than once a day | 169 (100.0%) | 92 (54.4%) | 77 (45.6%) |  |
| Unknown | 154 | 95 | 59 |  |
| Drinking in the Last 2 Years |  |  |  | <0.001 |
| No | 7,735 (100.0%) | 6,304 (81.5%) | 1,431 (18.5%) |  |
| Yes | 15,369 (100.0%) | 11,606 (75.5%) | 3,763 (24.5%) |  |
| Pre-pregnancy Healthcare Visit |  |  |  | <0.001 |
| No | 8,061 (100.0%) | 6,495 (80.6%) | 1,566 (19.4%) |  |
| Yes | 15,043 (100.0%) | 11,415 (75.9%) | 3,628 (24.1%) |  |
| Pre-pregnancy Checkup with Doctor |  |  |  | 0.2 |
| No | 6,721 (100.0%) | 5,249 (78.1%) | 1,472 (21.9%) |  |
| Yes | 16,383 (100.0%) | 12,661 (77.3%) | 3,722 (22.7%) |  |
| Pre-pregnancy Checkup with Obstetrician–Gynecologist |  |  |  | <0.001 |
| No | 6,564 (100.0%) | 4,818 (73.4%) | 1,746 (26.6%) |  |
| Yes | 16,540 (100.0%) | 13,092 (79.2%) | 3,448 (20.8%) |  |
| Pre-pregnancy Visit for Illness |  |  |  | 0.06 |
| No | 11,977 (100.0%) | 9,345 (78.0%) | 2,632 (22.0%) |  |
| Yes | 11,127 (100.0%) | 8,565 (77.0%) | 2,562 (23.0%) |  |
| Pre-pregnancy Visit for Injury |  |  |  | <0.001 |
| No | 13,896 (100.0%) | 10,653 (76.7%) | 3,243 (23.3%) |  |
| Yes | 9,208 (100.0%) | 7,257 (78.8%) | 1,951 (21.2%) |  |
| Pre-pregnancy Visit for Family Planning/Birth Control |  |  |  | <0.001 |
| No | 12,040 (100.0%) | 9,187 (76.3%) | 2,853 (23.7%) |  |
| Yes | 11,064 (100.0%) | 8,723 (78.8%) | 2,341 (21.2%) |  |
| Pre-pregnancy Visit for Depression or Anxiety |  |  |  | <0.001 |
| No | 12,154 (100.0%) | 10,288 (84.6%) | 1,866 (15.4%) |  |
| Yes | 10,950 (100.0%) | 7,622 (69.6%) | 3,328 (30.4%) |  |
| Pre-pregnancy Visit with Dentist |  |  |  | <0.001 |
| No | 6,501 (100.0%) | 4,627 (71.2%) | 1,874 (28.8%) |  |
| Yes | 16,603 (100.0%) | 13,283 (80.0%) | 3,320 (20.0%) |  |
| Pre-pregnancy Other Healthcare |  |  |  | <0.001 |
| No | 12,836 (100.0%) | 9,729 (75.8%) | 3,107 (24.2%) |  |
| Yes | 10,268 (100.0%) | 8,181 (79.7%) | 2,087 (20.3%) |  |
| During pre-pregnancy healthcare visits, a healthcare worker did: | | | | |
| Tell me to take a vitamin with folic acid |  |  |  | <0.001 |
| No | 10,465 (100.0%) | 7,840 (74.9%) | 2,625 (25.1%) |  |
| Yes | 12,639 (100.0%) | 10,070 (79.7%) | 2,569 (20.3%) |  |
| Talk to me about maintaining a healthy weight |  |  |  | 0.8 |
| No | 8,852 (100.0%) | 6,856 (77.5%) | 1,996 (22.5%) |  |
| Yes | 14,252 (100.0%) | 11,054 (77.6%) | 3,198 (22.4%) |  |
| Talk to me about controlling any medical conditions such as diabetes or high blood pressure |  |  |  | <0.001 |
| No | 12,119 (100.0%) | 9,286 (76.6%) | 2,833 (23.4%) |  |
| Yes | 10,985 (100.0%) | 8,624 (78.5%) | 2,361 (21.5%) |  |
| Talk to me about my desire to have or not have children |  |  |  | <0.001 |
| No | 8,821 (100.0%) | 6,637 (75.2%) | 2,184 (24.8%) |  |
| Yes | 14,283 (100.0%) | 11,273 (78.9%) | 3,010 (21.1%) |  |
| Talk to me about using birth control to prevent pregnancy |  |  |  | 0.02 |
| No | 7,837 (100.0%) | 6,143 (78.4%) | 1,694 (21.6%) |  |
| Yes | 15,267 (100.0%) | 11,767 (77.1%) | 3,500 (22.9%) |  |
| Talk to me about how I could improve my health before a pregnancy |  |  |  | <0.001 |
| No | 10,237 (100.0%) | 7,759 (75.8%) | 2,478 (24.2%) |  |
| Yes | 12,867 (100.0%) | 10,151 (78.9%) | 2,716 (21.1%) |  |
| Talk to me about sexually transmitted infections |  |  |  | 0.07 |
| No | 9,837 (100.0%) | 7,682 (78.1%) | 2,155 (21.9%) |  |
| Yes | 13,267 (100.0%) | 10,228 (77.1%) | 3,039 (22.9%) |  |
| Ask me if I was smoking cigarettes |  |  |  | <0.001 |
| No | 3,031 (100.0%) | 2,498 (82.4%) | 533 (17.6%) |  |
| Yes | 20,073 (100.0%) | 15,412 (76.8%) | 4,661 (23.2%) |  |
| Ask me if someone was hurting me emotionally or physically |  |  |  | <0.001 |
| No | 6,149 (100.0%) | 4,935 (80.3%) | 1,214 (19.7%) |  |
| Yes | 16,955 (100.0%) | 12,975 (76.5%) | 3,980 (23.5%) |  |
| Ask me if I was feeling down or depressed |  |  |  | <0.001 |
| No | 5,262 (100.0%) | 4,557 (86.6%) | 705 (13.4%) |  |
| Yes | 17,842 (100.0%) | 13,353 (74.8%) | 4,489 (25.2%) |  |
| Ask me about the kind of work I do |  |  |  | >0.9 |
| No | 5,108 (100.0%) | 3,962 (77.6%) | 1,146 (22.4%) |  |
| Yes | 17,996 (100.0%) | 13,948 (77.5%) | 4,048 (22.5%) |  |
| Test me for Human Immunodeficiency Virus (HIV) |  |  |  | 0.3 |
| No | 9,299 (100.0%) | 7,177 (77.2%) | 2,122 (22.8%) |  |
| Yes | 13,805 (100.0%) | 10,733 (77.7%) | 3,072 (22.3%) |  |
| Start of Prenatal Care in 1st Trimester |  |  |  | <0.001 |
| No | 2,946 (100.0%) | 2,225 (75.5%) | 721 (24.5%) |  |
| No prenatal care | 246 (100.0%) | 173 (70.3%) | 73 (29.7%) |  |
| Yes | 19,323 (100.0%) | 15,087 (78.1%) | 4,236 (21.9%) |  |
| Unknown | 589 | 425 | 164 |  |
| No. of Prenatal Care Visits | 11.1 (4.5) | 11.1 (4.3) | 10.9 (4.8) | <0.001 |
| Unknown | 493 | 374 | 119 |  |
| Kessner Index |  |  |  | <0.001 |
| 1. Unknown | 1,043 (100.0%) | 793 (76.0%) | 250 (24.0%) |  |
| 2. Inadequate | 1,597 (100.0%) | 1,169 (73.2%) | 428 (26.8%) |  |
| 3. Intermediate | 4,762 (100.0%) | 3,610 (75.8%) | 1,152 (24.2%) |  |
| 4. Adequate | 15,702 (100.0%) | 12,338 (78.6%) | 3,364 (21.4%) |  |
| Kotelchuck Index |  |  |  | <0.001 |
| 1. Inadequate | 2,991 (100.0%) | 2,227 (74.5%) | 764 (25.5%) |  |
| 2. Intermediate | 2,245 (100.0%) | 1,764 (78.6%) | 481 (21.4%) |  |
| 3. Adequate | 9,187 (100.0%) | 7,294 (79.4%) | 1,893 (20.6%) |  |
| 4. Adequate plus | 8,201 (100.0%) | 6,262 (76.4%) | 1,939 (23.6%) |  |
| Unknown | 480 | 363 | 117 |  |
| During Prenatal Care visits, a healthcare worker asked: | | | | |
| Ask if I knew how much weight I should gain during pregnancy |  |  |  | <0.001 |
| No | 10,033 (100.0%) | 7,662 (76.4%) | 2,371 (23.6%) |  |
| Yes | 13,071 (100.0%) | 10,248 (78.4%) | 2,823 (21.6%) |  |
| Ask if I was smoking cigarettes |  |  |  | 0.3 |
| No | 883 (100.0%) | 696 (78.8%) | 187 (21.2%) |  |
| Yes | 22,221 (100.0%) | 17,214 (77.5%) | 5,007 (22.5%) |  |
| Ask if I was drinking alcohol |  |  |  | 0.3 |
| No | 1,062 (100.0%) | 808 (76.1%) | 254 (23.9%) |  |
| Yes | 22,042 (100.0%) | 17,102 (77.6%) | 4,940 (22.4%) |  |
| Ask if I was taking any prescription medication |  |  |  | 0.7 |
| No | 1,011 (100.0%) | 788 (77.9%) | 223 (22.1%) |  |
| Yes | 22,093 (100.0%) | 17,122 (77.5%) | 4,971 (22.5%) |  |
| Ask if someone was hurting me emotionally or physically |  |  |  | 0.02 |
| No | 4,771 (100.0%) | 3,758 (78.8%) | 1,013 (21.2%) |  |
| Yes | 18,333 (100.0%) | 14,152 (77.2%) | 4,181 (22.8%) |  |
| Ask if I was feeling down or depressed |  |  |  | <0.001 |
| No | 3,474 (100.0%) | 2,852 (82.1%) | 622 (17.9%) |  |
| Yes | 19,630 (100.0%) | 15,058 (76.7%) | 4,572 (23.3%) |  |
| Ask if I was using drugs such as marijuana, cocaine, crack, or meth |  |  |  | <0.001 |
| No | 3,230 (100.0%) | 2,598 (80.4%) | 632 (19.6%) |  |
| Yes | 19,874 (100.0%) | 15,312 (77.0%) | 4,562 (23.0%) |  |
| Ask if I wanted to be tested for HIV |  |  |  | 0.3 |
| No | 8,441 (100.0%) | 6,510 (77.1%) | 1,931 (22.9%) |  |
| Yes | 14,663 (100.0%) | 11,400 (77.7%) | 3,263 (22.3%) |  |
| Ask if I planned to breastfeed my new baby |  |  |  | 0.2 |
| No | 1,622 (100.0%) | 1,277 (78.7%) | 345 (21.3%) |  |
| Yes | 21,482 (100.0%) | 16,633 (77.4%) | 4,849 (22.6%) |  |
| Ask if I planned to use birth control after my baby was born |  |  |  | 0.3 |
| No | 3,274 (100.0%) | 2,563 (78.3%) | 711 (21.7%) |  |
| Yes | 19,830 (100.0%) | 15,347 (77.4%) | 4,483 (22.6%) |  |
| Depression Before Pregnancy |  |  |  | <0.001 |
| No | 17,904 (100.0%) | 16,359 (91.4%) | 1,545 (8.63%) |  |
| Yes | 5,200 (100.0%) | 1,551 (29.8%) | 3,649 (70.2%) |  |
| ^1^n (%); Mean (SD)  ^2^Pearson’s Chi-squared test; Wilcoxon rank sum test | | | | |
