# Supplementary material for: Race/ethnicity, disability, and antenatal depression in the United States: population-level insights from machine learning
Source: Prev Med Rep. 2026 Mar 7;65:103437. doi: 10.1016/j.pmedr.2026.103437 (PMC12996995; doi:10.1016/j.pmedr.2026.103437)
Supplement: Supplementary file 4 — Appendix D [file mmc4.docx]

Appendix D. Association with Antenatal Depression by Maternal Race/Ethnicity and Disability Status Among Pregnant Women in 23 U.S. States and Jurisdictions, 2019 Pregnancy Risk Assessment Monitoring System

|  | **Non-Hispanic Black** | | | | **Non-Hispanic White** | | | |
| --- | --- | --- | --- | --- | --- | --- | --- | --- |
|  | **No Disability** N = 619^1^ | ***p*-value***^2^* | **At Least One Disability** N = 1,206^1^ | ***p*-value**^2^ | **No Disability** N = 1,018^1^ | ***p*-value***^2^* | **At Least One Disability** N = 2,351^1^ | ***p*-value**^2^ |
| Maternal Age | 27.1 (5.7) | <0.001 | 27.5 (6.0) | 0.01 | 27.5 (5.5) | <0.001 | 27.3 (5.6) | <0.001 |
| Marital Status |  | <0.001 |  | <0.001 |  | <0.001 |  | <0.001 |
| Married | 105 (7.52%) |  | 223 (25.1%) |  | 539 (10.7%) |  | 1,026 (27.7%) |  |
| Other | 514 (13.8%) |  | 983 (35.4%) |  | 479 (19.6%) |  | 1,325 (42.2%) |  |
| No. of Disabilities |  |  |  | <0.001 |  |  |  | <0.001 |
| 0 | 619 (12.1%) |  |  |  | 1,018 (13.6%) |  |  |  |
| 1 |  |  | 555 (26.0%) |  |  |  | 1,030 (26.9%) |  |
| 2 |  |  | 377 (38.2%) |  |  |  | 768 (39.4%) |  |
| 3 |  |  | 161 (45.4%) |  |  |  | 338 (47.1%) |  |
| 4 |  |  | 70 (61.9%) |  |  |  | 152 (58.0%) |  |
| 5 |  |  | 32 (68.1%) |  |  |  | 49 (69.0%) |  |
| 6 |  |  | 11 (37.9%) |  |  |  | 14 (70.0%) |  |
| Difficulty Seeing |  |  |  | 0.2 |  |  |  | 0.05 |
| No | 619 (12.1%) |  | 578 (32.0%) |  | 1,018 (13.6%) |  | 1,207 (33.3%) |  |
| Yes |  |  | 628 (33.8%) |  |  |  | 1,144 (35.5%) |  |
| Difficulty Seeing |  |  |  | 0.4 |  |  |  | <0.001 |
| 1 = No difficulty | 619 (12.1%) |  | 578 (32.0%) |  | 1,018 (13.6%) |  | 1,207 (33.3%) |  |
| 2 = Some difficulty |  |  | 529 (33.8%) |  |  |  | 1,023 (34.7%) |  |
| 3 = Lot of difficulty |  |  | 86 (35.0%) |  |  |  | 114 (46.2%) |  |
| 4 = Cannot do at all |  |  | 13 (26.5%) |  |  |  | 7 (31.8%) |  |
| Difficulty Hearing |  |  |  | 0.01 |  |  |  | <0.001 |
| No | 619 (12.1%) |  | 1,052 (32.2%) |  | 1,018 (13.6%) |  | 1,987 (33.3%) |  |
| Yes |  |  | 154 (38.9%) |  |  |  | 364 (40.9%) |  |
| Difficulty Hearing |  |  |  | 0.02 |  |  |  | <0.001 |
| 1 = No difficulty | 619 (12.1%) |  | 1,052 (32.2%) |  | 1,018 (13.6%) |  | 1,987 (33.3%) |  |
| 2 = Some difficulty |  |  | 129 (40.8%) |  |  |  | 311 (39.5%) |  |
| 3 = Lot of difficulty |  |  | 11 (31.4%) |  |  |  | 46 (56.1%) |  |
| 4 = Cannot do at all |  |  | 14 (31.1%) |  |  |  | 7 (36.8%) |  |
| Difficulty Walking |  |  |  | <0.001 |  |  |  | <0.001 |
| No | 619 (12.1%) |  | 941 (31.3%) |  | 1,018 (13.6%) |  | 1,883 (32.4%) |  |
| Yes |  |  | 265 (40.0%) |  |  |  | 468 (44.9%) |  |
| Difficulty Walking |  |  |  | <0.001 |  |  |  | <0.001 |
| 1 = No difficulty | 619 (12.1%) |  | 941 (31.3%) |  | 1,018 (13.6%) |  | 1,883 (32.4%) |  |
| 2 = Some difficulty |  |  | 214 (39.5%) |  |  |  | 411 (44.3%) |  |
| 3 = Lot of difficulty |  |  | 43 (45.3%) |  |  |  | 55 (50.5%) |  |
| 4 = Cannot do at all |  |  | 8 (30.8%) |  |  |  | 2 (33.3%) |  |
| Difficulty Remembering |  |  |  | <0.001 |  |  |  | <0.001 |
| No | 619 (12.1%) |  | 301 (22.3%) |  | 1,018 (13.6%) |  | 429 (23.6%) |  |
| Yes |  |  | 905 (39.1%) |  |  |  | 1,922 (38.2%) |  |
| Difficulty Remembering |  |  |  | <0.001 |  |  |  | <0.001 |
| 1 = No difficulty | 619 (12.1%) |  | 301 (22.3%) |  | 1,018 (13.6%) |  | 429 (23.6%) |  |
| 2 = Some difficulty |  |  | 711 (36.6%) |  |  |  | 1,457 (34.4%) |  |
| 3 = Lot of difficulty |  |  | 183 (52.7%) |  |  |  | 458 (58.6%) |  |
| 4 = Cannot do at all |  |  | 11 (39.3%) |  |  |  | 7 (50.0%) |  |
| Difficulty with Self-care |  |  |  | <0.001 |  |  |  | <0.001 |
| No | 619 (12.1%) |  | 1,090 (31.8%) |  | 1,018 (13.6%) |  | 2,068 (32.5%) |  |
| Yes |  |  | 116 (47.9%) |  |  |  | 283 (58.4%) |  |
| Difficulty with Self-care |  |  |  | <0.001 |  |  |  | <0.001 |
| 1 = No difficulty | 619 (12.1%) |  | 1,090 (31.8%) |  | 1,018 (13.6%) |  | 2,068 (32.5%) |  |
| 2 = Some difficulty |  |  | 94 (47.5%) |  |  |  | 251 (57.7%) |  |
| 3 = Lot of difficulty |  |  | 13 (65.0%) |  |  |  | 29 (64.4%) |  |
| 4 = Cannot do at all |  |  | 9 (37.5%) |  |  |  | 3 (60.0%) |  |
| Difficulty Communicating |  |  |  | <0.001 |  |  |  | <0.001 |
| No | 619 (12.1%) |  | 976 (31.4%) |  | 1,018 (13.6%) |  | 2,015 (33.0%) |  |
| Yes |  |  | 230 (40.9%) |  |  |  | 336 (45.8%) |  |
| Difficulty Communicating |  |  |  | <0.001 |  |  |  | <0.001 |
| 1 = No difficulty | 619 (12.1%) |  | 976 (31.4%) |  | 1,018 (13.6%) |  | 2,015 (33.0%) |  |
| 2 = Some difficulty |  |  | 182 (38.9%) |  |  |  | 291 (44.7%) |  |
| 3 = Lot of difficulty |  |  | 39 (55.7%) |  |  |  | 43 (55.1%) |  |
| 4 = Cannot do at all |  |  | 9 (37.5%) |  |  |  | 2 (40.0%) |  |
| Total Annual Income |  | - |  |  |  |  |  | - |
| 01. $0 to $16,000 | 241 (15.1%) |  | 541 (39.9%) |  | 238 (24.3%) |  | 668 (46.3%) |  |
| 02. $16,001 to $20,000 | 75 (13.7%) |  | 138 (33.8%) |  | 95 (20.4%) |  | 212 (38.0%) |  |
| 03. $20,001 to $24,000 | 36 (9.5%) |  | 90 (32.1%) |  | 60 (15.6%) |  | 188 (39.6%) |  |
| 04. $24,001 to $28,000 | 32 (11.2%) |  | 50 (23.5%) |  | 51 (17.2%) |  | 112 (35.3%) |  |
| 05. $28,001 to $32,000 | 32 (10.7%) |  | 58 (27.0%) |  | 55 (14.0%) |  | 161 (34.2%) |  |
| 06. $32,001 to $40,000 | 37 (9.9%) |  | 85 (32.8%) |  | 80 (13.4%) |  | 180 (32.3%) |  |
| 07. $40,001 to $48,000 | 26 (10.1%) |  | 31 (20.9%) |  | 72 (13.4%) |  | 135 (31.1%) |  |
| 08. $48,001 to $57,000 | 16 (7.3%) |  | 43 (26.5%) |  | 73 (10.6%) |  | 173 (31.3%) |  |
| 09. $57,001 to $60,000 | 15 (12.1%) |  | 20 (26.3%) |  | 51 (10.6%) |  | 103 (30.5%) |  |
| 10. $60,001 to $73,000 | 12 (7.1%) |  | 25 (22.1%) |  | 83 (8.7%) |  | 143 (22.8%) |  |
| 11. $73,001 to $85,000 | 9 (6.5%) |  | 17 (19.3%) |  | 86 (9.1%) |  | 150 (23.7%) |  |
| 12. $85,001 to $100,000 | 2 (18.2%) |  | 1 (33.3%) |  | 1 (14.3%) |  | 1 (33.3%) |  |
| 13. $100,001 to $120,000 | 0 (0%) |  | 1 (25.0%) |  | 1 (7.7%) |  | 1 (10.0%) |  |
| 14. $120,001+ | 1 (4.8%) |  | 2 (15.4%) |  | 9 (3.9%) |  | 7 (10.3%) |  |
| Unknown | 85 |  | 104 |  | 63 |  | 117 |  |
| Maternal Highest Degree |  |  |  | <0.001 |  | <0.001 |  | <0.001 |
| 1. < = 8th Grade | 1 (2.1%) |  | 18 (31.0%) |  | 10 (8.93%) |  | 23 (31.5%) |  |
| 2. 9-12th Grade | 120 (20.5%) |  | 166 (39.2%) |  | 110 (24.3%) |  | 282 (45.0%) |  |
| 3. High School Grad/GED | 230 (12.0%) |  | 451 (35.0%) |  | 323 (18.2%) |  | 807 (39.5%) |  |
| 4. Some College | 168 (12.6%) |  | 368 (32.9%) |  | 248 (15.8%) |  | 670 (37.3%) |  |
| 5. Associate Degree | 40 (9.3%) |  | 85 (30.1%) |  | 92 (10.9%) |  | 228 (32.1%) |  |
| 6. Bachelors Degree | 45 (7.8%) |  | 79 (22.4%) |  | 157 (8.6%) |  | 234 (20.3%) |  |
| 7. Masters Degree | 10 (5.6%) |  | 26 (25.2%) |  | 59 (8.7%) |  | 85 (24.5%) |  |
| 8. Doctorate/Professional Degree | 3 (8.6%) |  | 2 (12.5%) |  | 15 (8.2%) |  | 15 (21.4%) |  |
| Unknown | 2 |  | 11 |  | 4 |  | 7 |  |
| Special Supplemental Nutrition Program for Women, Infants, and Children During Pregnancy |  | 0.7 |  | <0.001 |  | <0.001 |  | <0.001 |
| No | 261 (11.9%) |  | 441 (29.6%) |  | 601 (11.2%) |  | 1,251 (29.6%) |  |
| Yes | 358 (12.3%) |  | 765 (35.1%) |  | 417 (19.8%) |  | 1,100 (41.9%) |  |
| Physical Abuse by Partner Before Pregnancy |  | <0.001 |  | <0.001 |  | <0.001 |  | <0.001 |
| No | 573 (11.5%) |  | 1,070 (31.1%) |  | 973 (13.2%) |  | 2,187 (33.3%) |  |
| Yes | 46 (36.2%) |  | 136 (58.6%) |  | 45 (37.5%) |  | 164 (57.7%) |  |
| Physical Abuse by Partner During Pregnancy |  | <0.001 |  | <0.001 |  | <0.001 |  | <0.001 |
| No | 571 (11.4%) |  | 1,096 (31.6%) |  | 974 (13.2%) |  | 2,210 (33.4%) |  |
| Yes | 48 (37.5%) |  | 110 (55.0%) |  | 44 (39.6%) |  | 141 (60.0%) |  |
| Physical Abuse by Ex-Partner Before Pregnancy |  | <0.001 |  | <0.001 |  | <0.001 |  | <0.001 |
| No | 574 (11.6%) |  | 1,083 (31.5%) |  | 960 (13.1%) |  | 2,136 (32.9%) |  |
| Yes | 45 (29.8%) |  | 123 (54.7%) |  | 58 (37.4%) |  | 215 (59.4%) |  |
| Physical Abuse by Ex-Partner During Pregnancy |  | <0.001 |  | <0.001 |  | <0.001 |  | <0.001 |
| No | 586 (11.7%) |  | 1,115 (31.9%) |  | 976 (13.3%) |  | 2,230 (33.5%) |  |
| Yes | 33 (28.0%) |  | 91 (52.3%) |  | 42 (40.0%) |  | 121 (60.2%) |  |
| No. of Loss of Pregnancy | 0.6 (1.0) | 0.01 | 0.7 (1.2) | 0.03 | 0.5 (1.0) | 0.01 | 0.6 (1.1) | <0.001 |
| Unknown | 2 |  | 2 |  | 1 |  | 2 |  |
| Infertility Treatment^3^ |  |  |  |  |  | 0.2 |  | <0.001 |
| No |  |  |  |  | 1,004 (13.7%) |  | 2,336 (34.5%) |  |
| Yes |  |  |  |  | 13 (9.49%) |  | 12 (15.4%) |  |
| Unknown |  |  |  |  | 1 |  | 3 |  |
| Diabetes Before Pregnancy |  | 0.00 |  | 0.01 |  | 0.01 |  | 0.8 |
| No | 579 (11.8%) |  | 1,121 (32.4%) |  | 970 (13.4%) |  | 2,253 (34.3%) |  |
| Yes | 40 (19.3%) |  | 85 (41.7%) |  | 48 (19.4%) |  | 98 (35.1%) |  |
| Hypertension Before Pregnancy |  | <0.001 |  | <0.001 |  | <0.001 |  | <0.001 |
| No | 527 (11.4%) |  | 992 (31.2%) |  | 929 (13.1%) |  | 2,131 (33.6%) |  |
| Yes | 92 (18.5%) |  | 214 (43.7%) |  | 89 (22.9%) |  | 220 (43.7%) |  |
| Diabetes During Pregnancy |  | <0.001 |  | <0.001 |  | <0.001 |  | <0.001 |
| No | 507 (10.9%) |  | 1,017 (31.4%) |  | 833 (12.4%) |  | 1,974 (32.7%) |  |
| Yes | 112 (23.4%) |  | 189 (44.6%) |  | 185 (25.2%) |  | 377 (46.7%) |  |
| Hypertension During Pregnancy |  | <0.001 |  | <0.001 |  | <0.001 |  | <0.001 |
| No | 400 (9.9%) |  | 769 (28.1%) |  | 701 (11.3%) |  | 1,689 (31.2%) |  |
| Yes | 219 (20.1%) |  | 437 (47.0%) |  | 317 (24.7%) |  | 662 (45.9%) |  |
| Body Mass Index Before Pregnancy^3^ |  |  |  |  | 28.2 (7.7) | <0.001 | 28.6 (8.0) | <0.001 |
| Unknown |  |  |  |  | 28 |  | 56 |  |
| Maternal Weight Gain (lbs)^3^ |  |  |  |  | 27.8 (16.5) | <0.001 | 29.2 (17.3) | 0.01 |
| Unknown |  |  |  |  | 16 |  | 48 |  |
| Pregnancy Intention |  | <0.001 |  | <0.001 |  | <0.001 |  | <0.001 |
| Later | 159 (12.7%) |  | 304 (31.2%) |  | 204 (17.0%) |  | 534 (38.6%) |  |
| Not sure | 186 (15.2%) |  | 349 (36.4%) |  | 214 (20.1%) |  | 555 (40.0%) |  |
| Not want | 103 (22.2%) |  | 223 (43.9%) |  | 92 (25.5%) |  | 263 (46.1%) |  |
| Sooner | 38 (9.2%) |  | 77 (25.7%) |  | 131 (11.8%) |  | 281 (30.8%) |  |
| Then | 126 (7.4%) |  | 229 (26.2%) |  | 349 (9.6%) |  | 675 (27.1%) |  |
| Unknown | 7 |  | 24 |  | 28 |  | 43 |  |
| No. Cigarettes Before Pregnancy | 1.3 (3.9) | <0.001 | 1.8 (5.9) | <0.001 | 2.9 (7.0) | <0.001 | 4.3 (8.7) | <0.001 |
| Unknown | 5 |  | 11 |  | 5 |  | 11 |  |
| No. Cigarettes in 1st Trimester | 0.9 (3.1) | <0.001 | 1.2 (4.8) | <0.001 | 1.9 (5.4) | <0.001 | 2.9 (6.7) | <0.001 |
| Unknown | 5 |  | 11 |  | 6 |  | 11 |  |
| No. Cigarettes in 2nd Trimester | 0.7 (2.6) | <0.001 | 0.9 (4.2) | <0.001 | 1.5 (5.1) | <0.001 | 2.2 (5.4) | <0.001 |
| Unknown | 5 |  | 10 |  | 4 |  | 11 |  |
| No. Cigarettes in 3rd Trimester | 0.6 (2.5) | <0.001 | 0.8 (4.0) | <0.001 | 1.4 (5.5) | <0.001 | 2.0 (5.6) | <0.001 |
| Unknown | 6 |  | 10 |  | 4 |  | 11 |  |
| E-Cigarettes Before Pregnancy |  | <0.001 |  | <0.001 |  | <0.001 |  | <0.001 |
| 1. Not use | 581 (11.7%) |  | 1,097 (31.7%) |  | 877 (12.5%) |  | 1,900 (31.8%) |  |
| 2. 1 day a week or less | 9 (31.0%) |  | 42 (57.5%) |  | 37 (31.4%) |  | 129 (54.7%) |  |
| 3. 2-6 days a week | 6 (24.0%) |  | 13 (48.1%) |  | 16 (28.6%) |  | 49 (48.5%) |  |
| 4. Once a day | 5 (31.3%) |  | 14 (58.3%) |  | 13 (26.5%) |  | 31 (41.9%) |  |
| 5. More than once a day | 8 (20.0%) |  | 21 (39.6%) |  | 54 (26.0%) |  | 208 (52.7%) |  |
| Unknown | 10 |  | 19 |  | 21 |  | 34 |  |
| E-Cigarettes During Pregnancy |  | 0.14 |  | <0.001 |  | 0.00 |  | <0.001 |
| 1. Not use | 610 (12.0%) |  | 1,158 (32.3%) |  | 973 (13.3%) |  | 2,161 (33.2%) |  |
| 2. 1 day a week or less | 1 (16.7%) |  | 15 (68.2%) |  | 11 (34.4%) |  | 56 (60.2%) |  |
| 3. 2-6 days a week | 2 (40.0%) |  | 7 (70.0%) |  | 6 (25.0%) |  | 28 (60.9%) |  |
| 4. Once a day | 1 (20.0%) |  | 6 (75.0%) |  | 4 (22.2%) |  | 19 (61.3%) |  |
| 5. More than once a day | 1 (20.0%) |  | 4 (36.4%) |  | 9 (22.5%) |  | 63 (55.8%) |  |
| Unknown | 4 |  | 16 |  | 15 |  | 24 |  |
| Drinking in the Last 2 Years |  | <0.001 |  | <0.001 |  | 0.00 |  | 0.3 |
| No | 242 (9.3%) |  | 399 (29.4%) |  | 248 (11.6%) |  | 542 (33.3%) |  |
| Yes | 377 (15.0%) |  | 807 (34.9%) |  | 770 (14.4%) |  | 1,809 (34.7%) |  |
| Pre-pregnancy Healthcare Visit |  | <0.001 |  | <0.001 |  | >0.9 |  | <0.001 |
| No | 235 (10.3%) |  | 391 (28.1%) |  | 322 (13.6%) |  | 618 (30.5%) |  |
| Yes | 384 (13.6%) |  | 815 (35.8%) |  | 696 (13.6%) |  | 1,733 (35.9%) |  |
| Checkup with Obstetrician–Gynecologist |  | 0.01 |  | 0.03 |  | 0.01 |  | <0.001 |
| No | 144 (14.4%) |  | 329 (35.8%) |  | 340 (15.3%) |  | 933 (38.6%) |  |
| Yes | 475 (11.5%) |  | 877 (31.9%) |  | 678 (12.9%) |  | 1,418 (32.0%) |  |
| Visit for Injury^3^ |  | 0.01 |  | <0.001 |  |  |  |  |
| No | 353 (13.3%) |  | 725 (35.3%) |  |  |  |  |  |
| Yes | 266 (10.8%) |  | 481 (29.9%) |  |  |  |  |  |
| Visit for Family Planning/Birth Control^3^ |  | 0.00 |  | 0.00 |  |  |  |  |
| No | 313 (13.8%) |  | 643 (35.4%) |  |  |  |  |  |
| Yes | 306 (10.8%) |  | 563 (30.4%) |  |  |  |  |  |
| Visit with Dentist |  | <0.001 |  | <0.001 |  | <0.001 |  | <0.001 |
| No | 195 (15.7%) |  | 419 (37.6%) |  | 324 (17.3%) |  | 936 (41.2%) |  |
| Yes | 424 (10.9%) |  | 787 (30.8%) |  | 694 (12.4%) |  | 1,415 (30.9%) |  |
| Other Healthcare |  | 0.00 |  | <0.001 |  | 0.2 |  | <0.001 |
| No | 344 (13.5%) |  | 722 (35.8%) |  | 606 (14.0%) |  | 1,435 (36.3%) |  |
| Yes | 275 (10.7%) |  | 484 (29.4%) |  | 412 (13.1%) |  | 916 (31.7%) |  |
| Tell me to take a vitamin with folic acid |  | 0.00 |  | 0.00 |  | 0.11 |  | <0.001 |
| No | 290 (13.8%) |  | 583 (35.4%) |  | 477 (14.3%) |  | 1,275 (37.6%) |  |
| Yes | 329 (10.9%) |  | 623 (30.8%) |  | 541 (13.1%) |  | 1,076 (31.1%) |  |
| Talk to me about controlling any medical conditions such as diabetes or high blood pressure^3^ |  | 0.5 |  | 0.06 |  |  |  |  |
| No | 253 (12.5%) |  | 554 (34.5%) |  |  |  |  |  |
| Yes | 366 (11.8%) |  | 652 (31.6%) |  |  |  |  |  |
| Talk to me about my desire to have or not have children |  | 0.08 |  | 0.01 |  | 0.03 |  | <0.001 |
| No | 233 (13.2%) |  | 494 (35.4%) |  | 416 (14.7%) |  | 1,041 (36.7%) |  |
| Yes | 386 (11.5%) |  | 712 (31.3%) |  | 602 (13.0%) |  | 1,310 (32.6%) |  |
| Talk to me about using birth control to prevent pregnancy^3^ |  |  |  |  |  | 0.01 |  | 0.00 |
| No |  |  |  |  | 375 (12.4%) |  | 857 (32.3%) |  |
| Yes |  |  |  |  | 643 (14.4%) |  | 1,494 (35.6%) |  |
| Talk to me about how I could improve my health before a pregnancy |  | 0.00 |  | 0.08 |  | >0.9 |  | 0.01 |
| No | 245 (14.1%) |  | 503 (34.5%) |  | 484 (13.6%) |  | 1,246 (35.8%) |  |
| Yes | 374 (11.1%) |  | 703 (31.8%) |  | 534 (13.7%) |  | 1,105 (32.8%) |  |
| Ask me if I was smoking cigarettes^3^ |  |  |  |  |  | <0.001 |  | <0.001 |
| No |  |  |  |  | 128 (10.6%) |  | 222 (25.9%) |  |
| Yes |  |  |  |  | 890 (14.2%) |  | 2,129 (35.5%) |  |
| Ask me if someone was hurting me emotionally or physically^3^ |  |  |  |  |  | <0.001 |  | <0.001 |
| No |  |  |  |  | 263 (11.1%) |  | 596 (30.1%) |  |
| Yes |  |  |  |  | 755 (14.8%) |  | 1,755 (36.1%) |  |
| Ask me if I was feeling down or depressed |  | 0.00 |  | <0.001 |  | <0.001 |  | <0.001 |
| No | 87 (9.3%) |  | 148 (22.2%) |  | 155 (7.2%) |  | 315 (20.7%) |  |
| Yes | 532 (12.7%) |  | 1,058 (35.3%) |  | 863 (16.2%) |  | 2,036 (38.2%) |  |
| Start of Prenatal Care in 1st Trimester |  | 0.2 |  | 0.2 |  | 0.6 |  | 0.01 |
| No prenatal care | 12 (18.2%) |  | 21 (34.4%) |  | 8 (15.1%) |  | 32 (48.5%) |  |
| No | 110 (12.8%) |  | 226 (36.1%) |  | 102 (14.6%) |  | 283 (37.2%) |  |
| Yes | 471 (11.7%) |  | 914 (32.2%) |  | 873 (13.3%) |  | 1,978 (33.6%) |  |
| Unknown | 26 |  | 45 |  | 35 |  | 58 |  |
| No. of Prenatal Care Visits^3^ |  |  |  |  | 11.2 (4.4) | 0.10 | 11.0 (4.6) | <0.001 |
| Unknown |  |  |  |  | 19 |  | 53 |  |
| Kessner Index |  | 0.07 |  | 0.3 |  | 0.02 |  | <0.001 |
| 1. Unknown | 33 (11.8%) |  | 56 (30.8%) |  | 43 (15.4%) |  | 118 (39.1%) |  |
| 2. Inadequate | 71 (15.2%) |  | 119 (34.3%) |  | 64 (17.4%) |  | 174 (41.8%) |  |
| 3. Intermediate | 165 (13.0%) |  | 310 (35.0%) |  | 201 (15.2%) |  | 476 (37.0%) |  |
| 4. Adequate | 350 (11.3%) |  | 721 (32.0%) |  | 710 (12.9%) |  | 1,583 (32.7%) |  |
| Kotelchuck Index^3^ |  |  |  |  |  | <0.001 |  | <0.001 |
| 1. Inadequate |  |  |  |  | 123 (16.3%) |  | 328 (41.3%) |  |
| 2. Intermediate |  |  |  |  | 84 (13.0%) |  | 201 (31.3%) |  |
| 3. Adequate |  |  |  |  | 408 (12.0%) |  | 900 (31.3%) |  |
| 4. Adequate plus |  |  |  |  | 383 (15.0%) |  | 872 (36.2%) |  |
| Unknown |  |  |  |  | 20 |  | 50 |  |
| Ask if I was using drugs such as marijuana, cocaine, crack, or meth^3^ |  |  |  |  |  | 0.02 |  | <0.001 |
| No |  |  |  |  | 139 (11.6%) |  | 289 (28.6%) |  |
| Yes |  |  |  |  | 879 (14.0%) |  | 2,062 (35.3%) |  |
| Ask if someone was hurting me emotionally or physically^3^ |  |  |  |  |  | 0.08 |  | <0.001 |
| No |  |  |  |  | 207 (12.4%) |  | 486 (30.0%) |  |
| Yes |  |  |  |  | 811 (14.0%) |  | 1,865 (35.7%) |  |
| Ask if I was feeling down or depressed |  | 0.2 |  | 0.01 |  | <0.001 |  | <0.001 |
| No | 61 (10.3%) |  | 131 (27.3%) |  | 124 (10.0%) |  | 306 (26.4%) |  |
| Yes | 558 (12.3%) |  | 1,075 (33.7%) |  | 894 (14.4%) |  | 2,045 (35.9%) |  |
| Depression Before Pregnancy |  | <0.001 |  | <0.001 |  | <0.001 |  | <0.001 |
| No | 327 (7.1%) |  | 388 (15.1%) |  | 338 (5.3%) |  | 492 (11.3%) |  |
| Yes | 292 (58.8%) |  | 818 (74.9%) |  | 680 (61.8%) |  | 1,859 (74.0%) |  |

^1^n (%); Mean (SD)

^2^Pearson’s Chi-squared test; Wilcoxon rank sum test

^3^Variables not significantly associated with antenatal depression and therefore excluded from subgroup analyses.
