# Supplementary material for: Race/ethnicity, disability, and antenatal depression in the United States: population-level insights from machine learning
Source: Prev Med Rep. 2026 Mar 7;65:103437. doi: 10.1016/j.pmedr.2026.103437 (PMC12996995; doi:10.1016/j.pmedr.2026.103437)
Supplement: Supplementary file 5 — Supplementary figures S1-S6 [file mmc5.docx]

Figure S1. Calibration plot for non-Hispanic Black women with at least one disability in 23 U.S. States and Jurisdictions, 2019 Pregnancy Risk Assessment Monitoring System


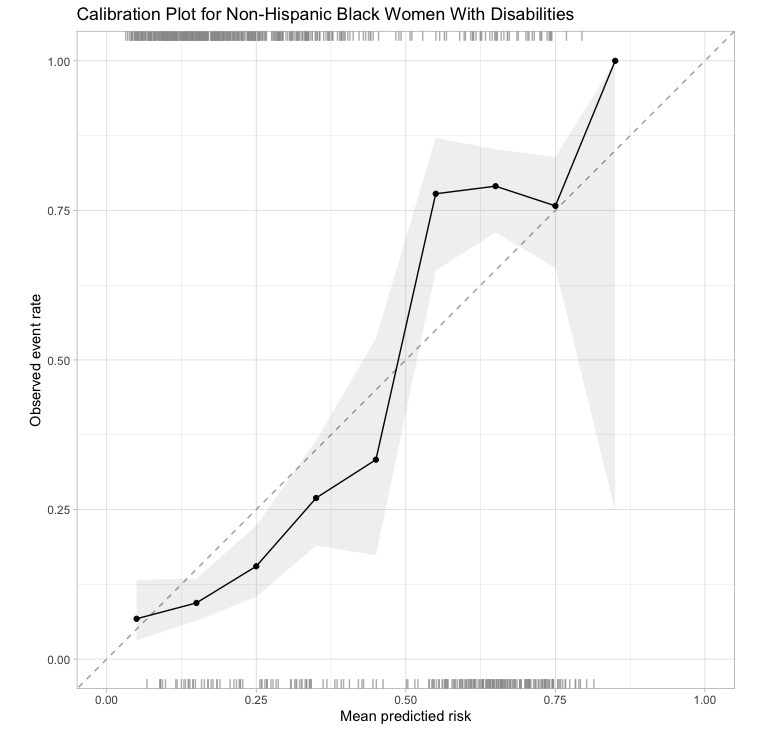


Figure S2. Calibration plot for non-Hispanic Black women without disability in 23 U.S. States and Jurisdictions, 2019 Pregnancy Risk Assessment Monitoring System

**
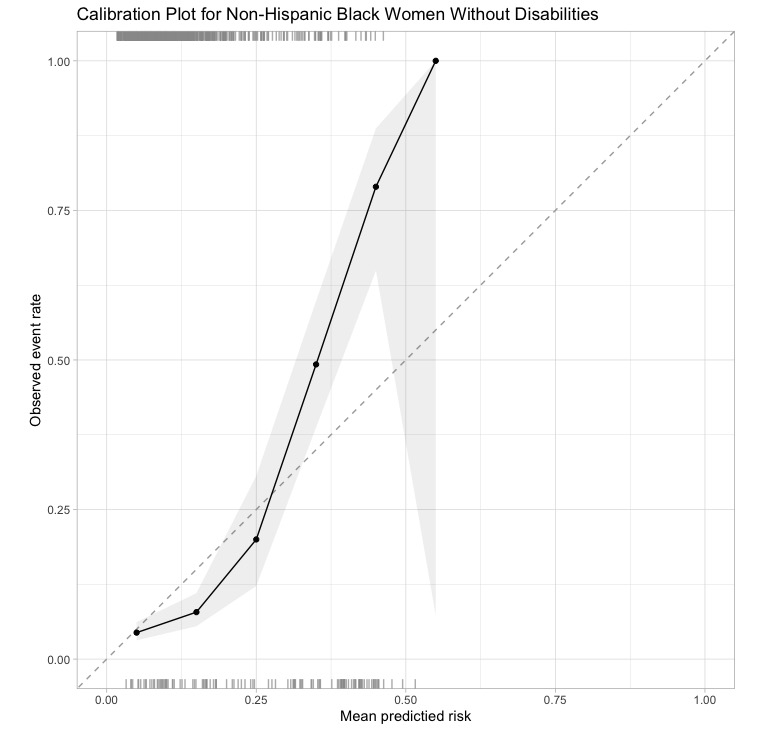
**

Figure S3. Calibration plot for non-Hispanic White women with at least one disability in 23 U.S. States and Jurisdictions, 2019 Pregnancy Risk Assessment Monitoring System

**
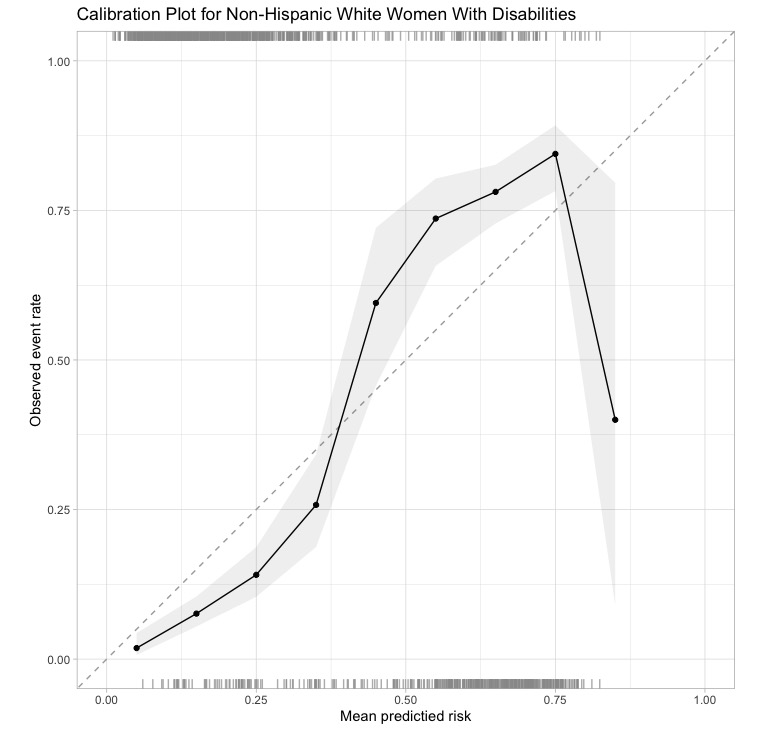
**

Figure S4. Calibration plot for non-Hispanic White women without disability in 23 U.S. States and Jurisdictions, 2019 Pregnancy Risk Assessment Monitoring System

**
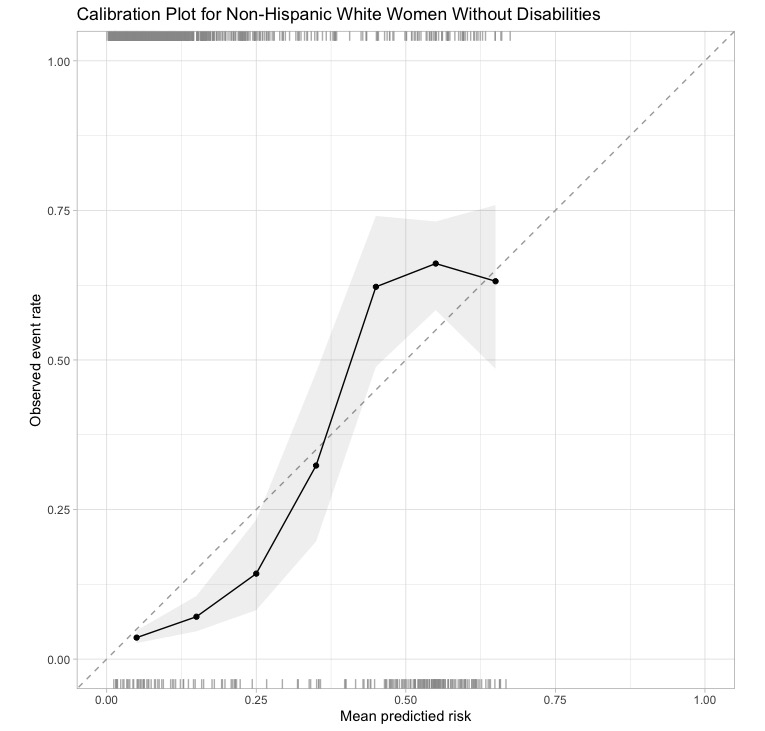
**

Figure S5. Top 20 predictors of antenatal depression among non-Hispanic Black women, pooled across disability statuses, in 23 U.S. States and Jurisdictions, 2019 Pregnancy Risk Assessment Monitoring System

**
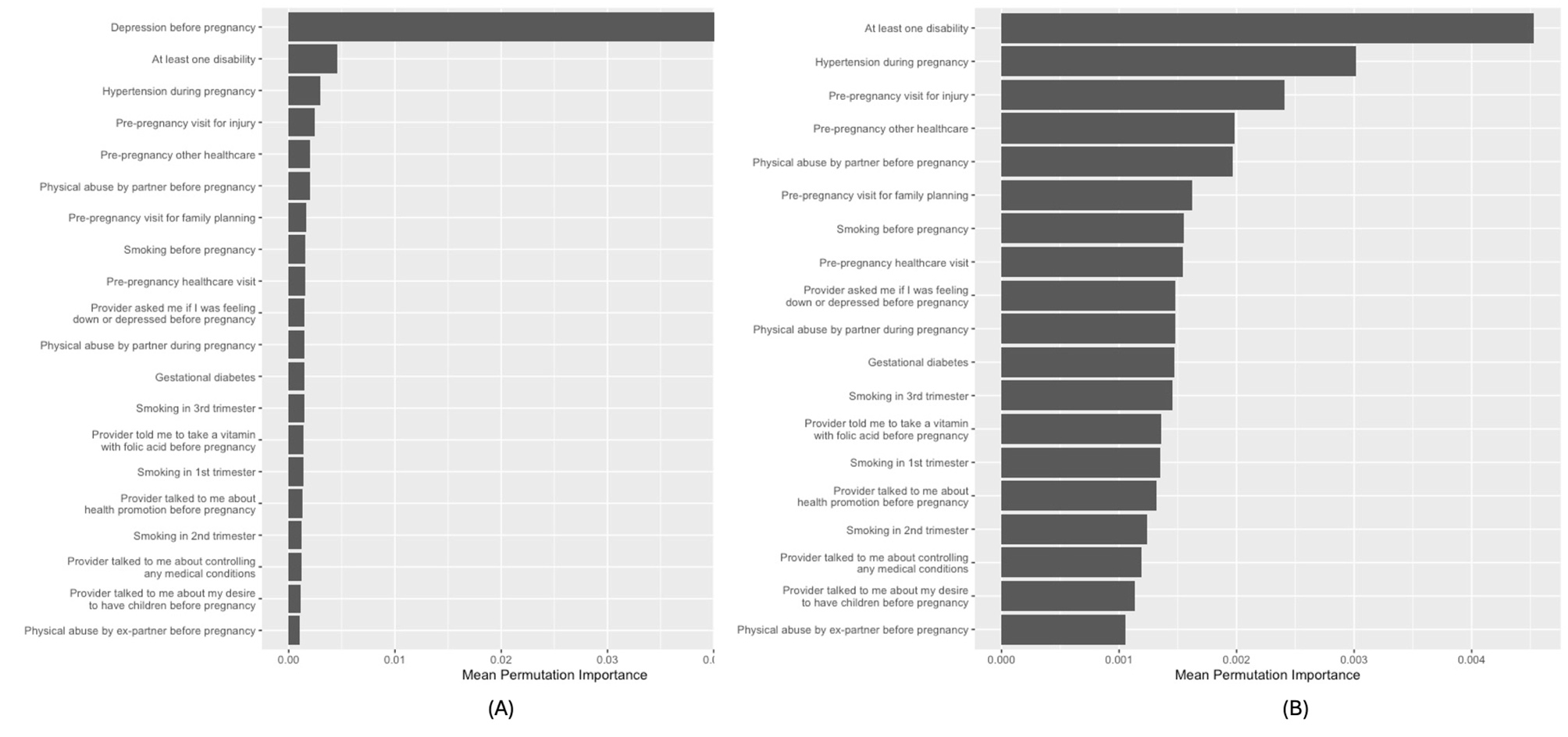
**

(A) Top 20 predictors including depression before pregnancy. Because depression before pregnancy had substantially higher importance, the scale is anchored to this variable, which compresses the relative magnitude of the remaining variables. (B) Top 19 predictors with depression before pregnancy removed to allow clearer visualization of differences in importance among the remaining variables. Variable importance values are unchanged across panels; only the scale differs.

Figure S6. Top 20 predictors of antenatal depression among non-Hispanic White women, pooled across disability statuses, in 23 U.S. States and Jurisdictions, 2019 Pregnancy Risk Assessment Monitoring System

**
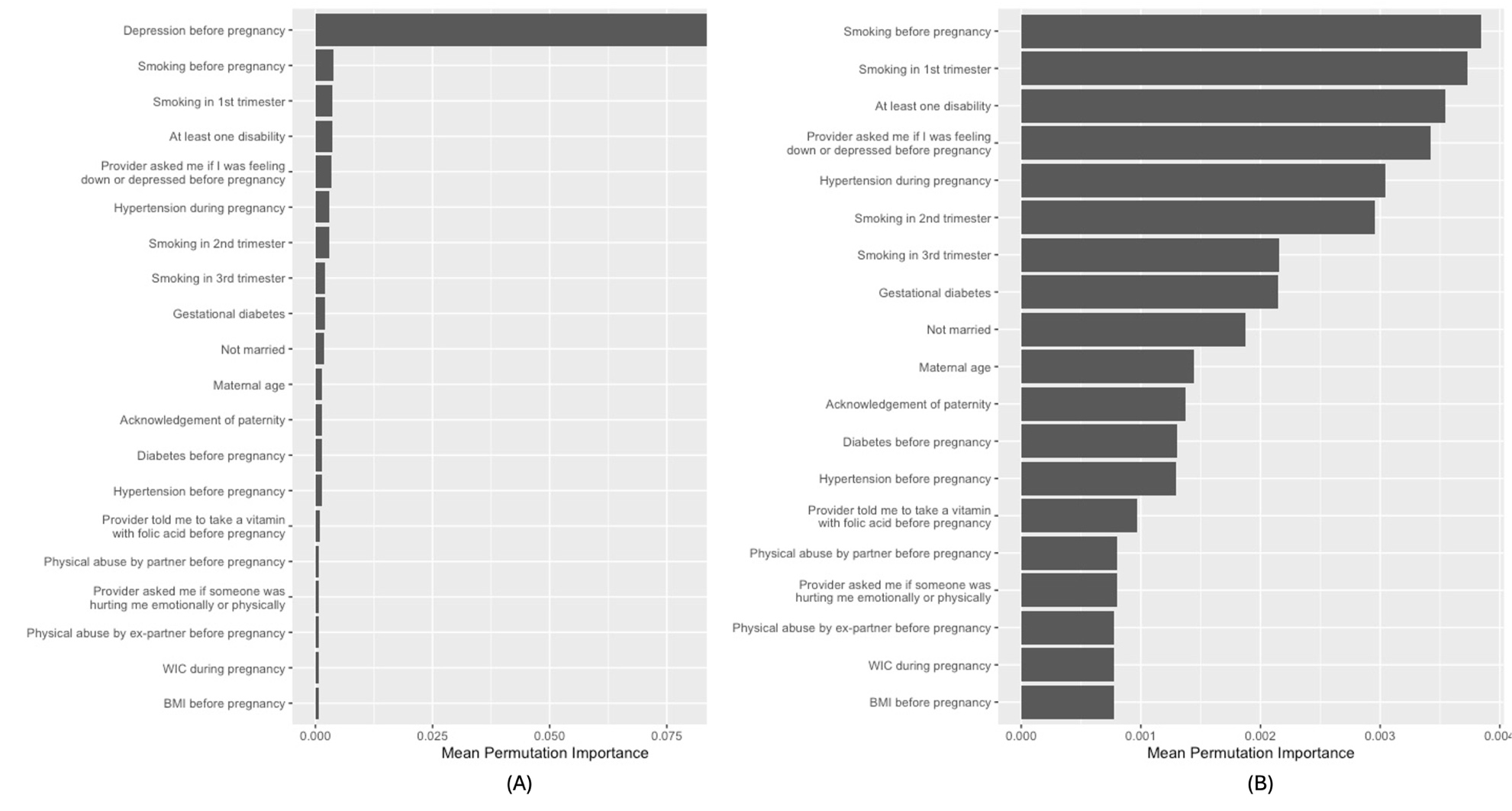
**

(A) Top 20 predictors including depression before pregnancy. Because depression before pregnancy had substantially higher importance, the scale is anchored to this variable, which compresses the relative magnitude of the remaining variables. (B) Top 19 predictors with depression before pregnancy removed to allow clearer visualization of differences in importance among the remaining variables. Variable importance values are unchanged across panels; only the scale differs.
